# Preserving nutrient content in red cabbage juice powder via foam‐mat hybrid microwave drying: Application in fortified functional pancakes

**DOI:** 10.1002/fsn3.3847

**Published:** 2023-11-20

**Authors:** Muhammed Mustafa Ozcelik, Sedef Aydin, Ebru Aydin, Gulcan Ozkan

**Affiliations:** ^1^ Department of Food Engineering, Faculty of Engineering and Natural Sciences Suleyman Demirel University Isparta Turkey

**Keywords:** *Brassica oleracea*, drying characteristics, hybrid‐drying system, response surface methodology

## Abstract

Red cabbage, a highly nutritious cool‐season cruciferous vegetable, is rich in anthocyanins; however, the instability of anthocyanins during processing and storage poses challenges. This study aimed to optimize the foam‐mat drying process of red cabbage juice (RCJ) with a high anthocyanin content using a hybrid microwave hot air‐drying system (MW‐HAD) as a dehydration method compared to conventional techniques (HAD) using response surface methodology (RSM). Additionally, the produced red cabbage juice powder (RCJP) was used to enrich the pancake formulation. The developed model exhibited a high degree of reliability with optimal conditions and was determined for microwave power, temperature, foaming agent carboxymethylcellulose (CMC), and egg white protein (EWP) as 360 W, 60°C, 0.3%, and 1.2%, respectively. Moisture content (%) was decreased from 93.47 to 8.62 at optimum process conditions. In comparison to the control (60°C), foam mat drying with the MW‐HAD hybrid system reduced the drying time (DT) by more than 90.9% due to the higher drying rate, while many physicochemical properties, especially total anthocyanin content (TAC), were better preserved. Utilization of RCJP in the production of anthocyanin‐rich functional pancakes resulted in enhanced nutritional qualities compared to control pancakes with increased protein (35.07%), total phenolic (75.8%), dietary fiber (82.9%), and anthocyanin content (100%). In conclusion, MW‐HAD demonstrates significant potential as a promising drying method to reduce the DT and preserve the physicochemical properties of RCJP. Furthermore, the application of the optimized RCJP in anthocyanin‐rich functional pancakes highlights improved nutritional qualities, making a substantial contribution to the advancement of functional foods.

## Graphical abstract

A hybrid microwave hot air‐drying system (MW‐HAD) was employed as the dehydration method, and the effectiveness of this approach was compared to conventional techniques. Additionally, the study aimed to assess the applicability of the optimized red cabbage juice powder (RCJP) in the production of functional pancakes.

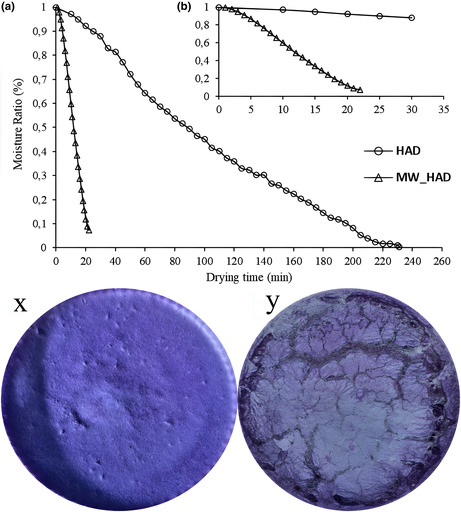

## 1 INTRODUCTION

Red cabbage (*Brassica oleracea* var. *capitata f. rubra*) features substantial red/purple leaves, grown in spring and harvested in autumn (Özbakir Özer et al., 2021), typically consumed as coleslaw, salad, and drink (Ávila et al., 2023). Red cabbage is highly nutritious and rich in bioactive components such as minerals, vitamins, oligosaccharides, anthocyanins, and flavonols (Drozdowska et al., 2020). The bioactive components of red cabbage that promote health activities are especially anthocyanins, vitamins A, B, C, K, and phenolics (Chauhan et al., 2016; Shankar et al., 2019). Anthocyanins are water‐soluble natural pigments that change between red, blue, and purple depending on pH (Drozdowska et al., 2020), and cyanidin‐3‐diglycoside‐5‐glycoside is the major anthocyanin fraction of red cabbage (Ghareaghajlou et al., 2022). Red cabbage is also high in soluble and insoluble fiber (Chauhan et al., 2016). Many studies conducted on red cabbage extract reveal its biological activities such as antibacterial, anticancer, antidiabetic, anti‐inflammatory, analgesic, and these vegetables act as powerful modulators for the innate immune response system (Buko et al., 2018; Shankar et al., 2019; Zayed et al., 2022).

Drying is the most popular technique for food preservation, which involves the removal of moisture to extend the shelf life of food products and help prevent microbial and enzymatic deterioration (Nisoa et al., 2021). Among traditional drying methods, sun and shade drying are the most commonly used drying techniques worldwide, as their applications are simple and low‐cost. However, the disadvantages of the solar drying process include its low drying efficiency, susceptibility to weather conditions significantly affecting the process, and vulnerability to contamination (Ai et al., 2022). The high drying temperature and long drying time of hot air convective drying, which is the most widely used among these methods, can cause significant deterioration in product quality, including changes in color, flavor, surface characteristics, and nutritional content. Consequently, the need for alternative techniques such as microwave drying has emerged (Kılıç & Çınar, 2019; Roknul Azam et al., 2019). Microwaves are based on dielectric heating by electromagnetic waves and offer advantages such as lower drying temperatures, faster drying speeds, more uniform energy distribution throughout the material, shorter drying times, and better quality of the final product (Bagheri, 2020; Nisoa et al., 2021). The disadvantage is the cracked temperature age of foodstuff, which is exceptionally affected by the microwave condition, and the moisture accumulating on the food surface (Ozcan‐Sinir et al., 2019). To overcome these problems, optimizing microwave power and microwave dryers require a hot air (HAD) dryer that serves as an additional heating source (Abbaspour‐Gilandeh et al., 2021). There are different artificial drying methods, and foam‐mat drying with applications such as foam freeze drying, foam shower drying, foam vacuum drying, and microwave‐assisted foam drying is an effective and cost‐effective procedure (Kumar et al., 2022; Qadri & Srivastava, 2021). Thus, microwave hot air‐drying system (MW‐HAD) enabled more consistent temperature distribution and surface moisture evacuation. Because of their fast drying times and intense heat treatment, these revolutionary drying systems offer the advantage of reducing quality degradation when used at optimum values (de Souza et al., 2022; Hnin et al., 2018; Zielinska et al., 2020). Microwave‐assisted foam drying (MAFD) research has been found in the literature, particularly in recent years. MAFD was used to dry yogurt, tomatoes, papaya, blueberry, and blue honeysuckle berry (Gao et al., 2022; Qadri et al., 2020; Sun et al., 2020; Yüksel, 2021). Foam‐mat drying, on the other hand, is a drying procedure used for drying semi‐liquid and liquid products rich in phytochemicals, sensitive to heat, and sticky (Kumar et al., 2022). According to previous studies, egg white powder is the most commonly used foaming agent, and carboxymethyl cellulose is the most commonly utilized foam stabilizer that employs foam‐mat drying methods (Çalışkan Koç et al., 2022). In this method, first, the liquid or semi‐liquid food to be dried is converted into foam with a foaming agent or surfactant with foam stabilizer properties and then convectively dried using hot air (Kumar et al., 2022; Maciel et al., 2022; Shaari et al., 2018). In addition, there are few foam‐mat drying studies in the literature with microwave freeze drying and microwave vacuum drying hybrid drying systems (Ozcelik et al., 2019; Sramek et al., 2015). Foam‐mat drying with MW‐HAD is a novel drying technique, and limited experiments have been discovered in the literature (Qadri et al., 2020; Qadri & Srivastava, 2017, 2021). In these studies, tomato, papaya, and guava were used as materials. The red cabbage foam‐mat drying study has not been reported. Also, no studies on red cabbage drying utilizing hybrid MW‐HAD have been published in the literature.

Response surface methodology (RSM) is a mathematical and statistical approach for efficient experimental design and is widely used for reducing the number of experiments, factors, and their levels (Chelladurai et al., 2020). Also, it's an effective method for modeling and optimizing of several variables and their interactions with one or more response variables (Ozcelik et al., 2022). The Box–Behnken design (BBD) is a simpler and more effective type of RSM than other three‐level factorial designs in which independent variables are tested at three levels to create the response model and fit the output (expected response) into a quadratic model (LaPanse et al., 2021; Martín‐García et al., 2020). Foam‐mat drying optimization with MW‐HAD using RSM is very scarce in the literature (Aghilinategh et al., 2015; Varhan et al., 2019). The red cabbage foam‐mat drying study and the optimization of drying conditions with RSM have not been found in the literature.

In this study, the objective was to investigate the drying of red cabbage juice using a foam‐mat drying process with hybrid microwave and hot air heating, utilizing different mixtures of carboxymethylcellulose (CMC) and egg white protein (EWP) as foaming agents. The study focused on evaluating the impact of the foaming agents and drying device parameters on the drying time and various physicochemical properties of the dried red cabbage juice powder (RCJP), including bulk density, color, browning index (BI), pH, °Bx, water activity, moisture content (MC), total anthocyanin content (TAC), ascorbic acid content (AAC), total phenolic content (TPC), and total antioxidant capacity (TEAC). The growing interest in the nutritional and functional properties of red cabbage has generated the exploration of alternative functional flours and their potential food applications. Therefore, another objective of this study was to develop a functional pancake formulation using RCJP as a substitute for conventional flour, enriched with egg white protein and CMC as a foaming agent.

## 2 MATERIALS AND METHODS

### 2.1 Material

The fresh red cabbages were collected based on their color and maturity from the local market in Isparta, Turkey. Also, the size, shape, harvest time, and visual look of each cabbage were considered.

### 2.2 Experimental design: Optimization of foaming agents and drying

Response surface methodology and Box–Behnken experimental design were used for determining optimal foaming agent conditions for fresh red cabbage juice. Based on the preliminary test results, the minimum (−1) and maximum (1) factor levels of the independent variables were defined as MW power (180–360 W), CMC (0.3% and 1.2%), and EWP (2% and 10%) concentrations and consisted of 15 experiments (Table 2). RSM was performed using Minitab® Statistical Software (Minitab® Inc., State College, USA), and model adequacy and regression tests were evaluated by adjusted *R* ^2^, predicted *R* ^2^, and lack of fit value. All runs were randomized, and six experimental responses (dependent variables) were presented (moisture, water activity, BI, anthocyanin content, ascorbic acid content, and drying time). The experimental data was used to fit a second‐order polynomial model in order to determine the regression coefficients (*β*). RSM analysis employed a generalized second‐order polynomial model, represented by Equation 1.

(1) $$Z=\beta_{0}+\sum_{i=1}^{3}\beta_{i}X_{i}+\sum_{i=1}^{3}\beta_{\mathrm{ii}}X_{i}^{2}+\sum_{i=1}^{2}\sum_{j=i+1}^{3}\beta_{\mathrm{ij}}X_{i}X_{j}$$

where *Z* is the dependent variable, *X* is the independent variable, and the constant coefficient is defined as *β*_0_ for intercept, *β*_*i*_ for linear, *β*_*ii*_ for quadratic, and *β*_*ij*_ for two‐factor interaction coefficient. Fresh red cabbage juice thick foam was spread into a circular microwave tray (23.5 cm diameter × 5 cm height) and dried between 0.5 and 1.6 cm thick foam in the microwave (MEZ58002743, LG, Korea) based on the optimization experiments. In the MW‐HAD system, the HAD temperature was set at a constant 60°C in all drying trials. Each 60 s, the tray was weighed manually and checked to see the drying process of the foamed fresh red cabbage juice.

As a positive control, a traditional convection hot air‐drying technique (Tk‐Lab model, EKSİS Machinery, Isparta, Turkey) was used to dry foam‐mat red cabbage juice at 60°C, and then the same dependent variables were compared. After drying (*a*_w_ ≤ 0.5), the flakes of the red cabbage juice were scrapped, grinded into powder, and passed through a 0.5 mm sieve. The powder was quickly kept in vacuum pack bags and stored in the refrigerator. In addition, the drying under optimized conditions Bx°, pH, total acidity (%), TPC, TEAC, bulk density, water absorption, solubility index, and swelling power were analyzed. Three replicates were performed in each analysis.

#### 2.2.1 Preparation of red cabbage juice

After removing the outer leaves and surface cleaning of fresh cabbage, the cabbage juice was extracted by grinding in a cold press juicer (HR‐1861, Philips, Eindhoven, Netherlands) and filtered through coarse filter paper. Subsequently, to create the foam of red cabbage juice, various concentrations of EWP and CMC were added to 150 mL of juice. The most suitable ratio of the foaming agents was identified through the utilization of the RSM‐Box Behnken experimental design.

#### 2.2.2 Preparation and drying of foam

Following the preliminary trials, it was decided that 150 g of red cabbage juice was sufficient for each experiment to be used in the forthcoming analyses. Different proportions of the foaming agents (Table 1) were mixed in 150 mL of fresh red cabbage juice using a hand mixer (the speed was increased gradually) for 5 min/until the thick foam appeared.

**TABLE 1:** Physicochemical properties of fresh red cabbage juice.

Analyze	Fresh cabbage juice
MC	93.47 ± 1.10
WA	0.92 ± 0.01
pH	6.16 ± 2.03
SSC	4.98 ± 0.21
TA	0.31 ± 0.02
L*	23.03 ± 0.07
a*	1.69 ± 0.17
b*	−2.22 ± 0.16
C*	2.80 ± 0.23
h°	307.16 ± 1.44
TAC	34.88 ± 0.11
AAC	32.6 ± 0.10
TEAC	1581.67 ± 7.20
TPC	21.64 ± 0.21

Abbreviations: AAC, ascorbic acid content (mg/100 g wb); *L*, *a*, *b*, *C*, h°, color values; MC, moisture content (%); SSC, total soluble solid content; TA, titration acidity (%); TAC, total anthocyanin content (mg cyanidin3g/100 g wb); TEAC, μmol T.E/100 g wb; TPC, total phenolic compound (mg GAE/100 g wb); WA, water activity (*a*_w_).

#### 2.2.3 Preparation of pancakes

Ingredients including flour (60 g), milk (50 mL), baking powder (2.7 g), salt (2.5 g), xanthan gum (0.25 g), and guar gum (0.25 g) were determined by preliminary tests and used to make the standard pancake dough. RCJP (20 g) replaced the equal weight of wheat flour pancakes by the weight of total flour in the mix. Then, the cooking pan was set on medium heat and fried for 1 min on both sides using 1 tablespoon of dough. With the amount of mixture used, 14 pancakes were produced, and 1 pancake had an average weight of 20 g.

### 2.3 Drying characteristics

Drying characteristics curves were created by plotting moisture content versus drying time. Equation 2 was used to determine the moisture ratio (MR) expression (Jeevarathinam et al., 2021).

(2) $$\mathrm{MR}=\frac{M-\mathrm{Me}}{\mathrm{Mi}-\mathrm{Me}}$$

where MR is the moisture ratio, *M* is the moisture content at any time *t* (% db), Me is the equilibrium moisture content (EMC) (% db), and Mi is the initial moisture content of the material (% db).

The drying rate was determined using Equation 3 (Jeevarathinam et al., 2021).

(3) $$\mathrm{DR}=\frac{M_{t+\mathrm{dt}}-M_{t}}{\mathrm{dt}}$$

where DR is the drying rate (g water/(g dry matter × h)), and *M*_*t*_ and *M*_*t* + *dt*_ are the moisture contents (db) at measuring time *d* and *t* + *dt*.

### 2.4 Foam capacity (FC) and foam stability (FS)

The values for FC and FS were calculated with a slight modification based on the method outlined by Ahmad et al. (2015). Twenty‐five milliliter of fresh red cabbage juice at 25 ± 2°C and foaming agents are mixed slowly with a spatula in a deep round bowl, and then mixed using an electrical mixer (Master mix, Tefal, France) for 5 min (with increasing speed gradually) until a thick foam is formed. The foam was transferred to a 100‐mL measuring cylinder, and after 30 s, the volume of the foam was measured. One hour after whipping, the foam volume was measured to calculate (FS) as a percentage of the initial foam volume. FC and FS are given in Equations 4 and 5, respectively.

(4) $$\mathrm{FC}=\frac{a-b}{a}\times 100$$

(5) $$\mathrm{FS}=\frac{x}{y}\times 100$$

where *a* represents the volume after mix, *b* represents the volume before mix, *x* is the foam volume after standing 60 min, and *y* is the initial foam volume.

### 2.5 Bulk density, Carr index, and Hausner ratio of foam‐mat‐dried powder

In order to determine the bulk density of fruit juice powders, the produced powder will be transferred into a measuring cylinder with a volume of 10 mL without pressure. The bulk density value (*dx*) will be determined by dividing the mass of the powder by its volume. The compressed density (*dy*) will be analyzed by tapping the measuring cylinder containing the powder on a hard surface 180 times. The compressed density value will be calculated by dividing the mass (*m*) of the powder by the volume record after tapping (Tatar & Kahyaoglu, 2015). Carr index (CI) and Hausner ratio (HR) values will be determined according to Equation 6 using bulk density and compressed density values (Goyal et al., 2015). The calculated data will provide information about the flow properties of powder products.

(6) $$\begin{array}{c} \mathrm{CI}=\left(1-\frac{\mathrm{dx}}{\mathrm{dy}}\right)\times 100\\ \mathrm{HR}=\left(\frac{\mathrm{dx}}{\mathrm{dy}}\right) \end{array}$$

### 2.6 Water absorption, solubility index, and swelling power of foam‐mat‐dried powder

In summary, a 50.0 mg powder sample (±0.1 mg) was mixed with 1 mL of distilled water, heated at 90°C for 15 min, and then cooled with ice. The resulting paste was centrifuged at 3000 × *g* and 4°C for 10 min, and the dry solids were obtained by evaporating the supernatant overnight at 110°C. Three replicates were prepared for each sample, and the water solubility index (WSI), water absorption index (WAI), and swelling power (SP) were determined following (de la Hera et al., 2013) method.

### 2.7 Moisture, water activity (*a*_w_), and total soluble solid content (SSC) of foam‐mat‐dried powder and pancakes

To evaluate moisture content, 3–4 g of sample were weighed and quantified at 105°C using a moisture analyzer (DBS 60‐3, Kern & Sohn GmbH, Germany). At 25°C, the water activity (*a*_w_) of the sample was measured using an electronic *a*_w_ device, the Thermoconsanter TH 200 (Novasina, Axair Ltd., Pfäffikon, Switzerland). These methods were adopted from Aydin et al. (2023).

### 2.8 Color parameters and browning index (B) of foam‐mat‐dried powder and pancakes

To determine the color of fresh and foam‐mat‐dried red cabbage powder, colorimeter (NH310 High‐Quality Portable Colorimeter, Shenzhen 3NH Technology LTD, China) was utilized, and the BI was calculated based on the previously published study (Ozcelik et al., 2022).

### 2.9 pH and acidity of cabbage juice and foam‐mat‐dried powder

The pH of both fresh red cabbage juice and foam‐mat‐dried red cabbage powder samples was measured using a pH meter (Sevencompact pH meter, Mettler‐Toledo, Greifensee, Switzerland). To determine the acidity (%) of both the fresh red cabbage juice and foam‐mat‐dried red cabbage powders, 5 g of each sample were weighed. Approximately 100 mL of distilled water was added to each sample, and the mixtures were homogenized using an Ultra Turrax homogenizer (T‐18 basic, Ika, Staufen, Germany) for 1 min. The resulting mixtures were then filtered. The obtained filtrates were titrated with a 0.1 N NaOH solution until the pH reached 8.1. The acidity was expressed as a chlorogenic acid equivalent.

### 2.10 Ascorbic acid content and ABTS radical scavenging activity of foam‐mat‐dried powder and pancakes

The methodology in the publication by Ozcelik et al. (2022) was employed to assess the levels of ascorbic acid and ABTS content in the samples. To quantify ascorbic acid content, the samples were combined with a 4.5% meta‐phosphoric acid solution at a ratio of 1:10 (g/mL), followed by centrifugation at 4000 rpm for 10 min at a temperature of 4°C. Subsequently, the resulting supernatant was filtered through a 0.45 μm Millipore filter, and 20 μL of the filtrate was introduced into an HPLC system (Agilent, 1260 infinity, Santa Clara, CA, USA) equipped with a diode array detector (DAD, Agilent, 1260 MWD VL, Santa Clara, CA, USA) and a C18 column (250 × 4.6 mm, ID: 5 μm ACE, Aberdeen, Scotland).

For the assessment of radical scavenging activity, a 7 mM ABTS solution containing 2.45 mM potassium persulfate was prepared and allowed to stand for 12–16 h, leading to the generation of the ABTS^·+^ radical. Subsequently, 10 μL of the appropriately diluted extract was introduced into 1 mL of the ABTS^·+^ solution, followed by vortexing for 10 s, and the alteration in color was monitored by measuring the absorbance at 734 nm using a spectrophotometer.

### 2.11 Anthocyanins and total phenolic composition of foam‐mat‐dried powder and pancakes by HPLC‐DAD

The anthocyanin and phenolic content of the samples were determined using a modified version of the procedure described by Kelebek and Selli (2011) and analyzed using an HPLC‐DAD system (Agilent, 1260 infinity, Santa Clara, CA, USA). In summary, the samples were ground, and 0.8 grams of the prepared sample were mixed with 25 mL of an extraction solution composed of acetonitrile, water, and acetic acid in a ratio of 70:29.5:0.5 (v/v/v). The mixture was homogenized using an Ultra Turrax homogenizer (T‐18 basic, Ika, Staufen, Germany) for 1 min. Subsequently, the sample was subjected to sonication for 15 min in an ice‐cooled ultrasonic water bath. Acetone was then evaporated from the solution under vacuum conditions at 35°C using an evaporator. The remaining aqueous residue was filtered through No. 1 Whatman filter paper and loaded onto an activated Sep‐Pack C‐18 solid‐phase extraction cartridge (Agilent, Bond Elut, C18, USA). First, water‐soluble compounds were removed by washing the cartridge with 8 mL of water, followed by the washing of the retained phenolic compounds with 8 mL of methanol. The methanol was completely evaporated under vacuum at 35°C, resulting in a dry extract that was then redissolved in 1 mL of the extraction solution. The resulting solution was subsequently filtered through a 0.45 μm nylon (PA) filter and subjected to analysis by HPLC. The separation of compounds was achieved using a C18 column (ACE, Aberdeen, UK) with dimensions of 4.6 mm i.d. × 250 mm and a particle size of 5.0 μm, along with a guard column (Agilent, Eclipse XDB C18, USA) of the same granulometry (4.6 mm i.d. × 10 cm, 5.0 μm). The analysis was performed at a temperature of 25°C with an injection volume of 20 μL, a flow rate of 0.5 mL/min, and detection at 520 nm wavelength for anthocyanins and 280 nm for phenolics. The total runtime of the analysis was 125 min. Mobile phase A: purified (HPLC grade) water/acetic acid (97:3; v/v), mobile phase B: methanol, HPLC flow rates: 0–5 min B: 7%, B: 0%–20% 27 min, B: 25% to 12 min, B: 30% to 12 min, B: 33% to 29 min, B: 42% to 5 min, B: 50% to 5 min, B: 70% to 5 min, B: It was applied in 5 min to 80%, B: 5 min to 100%, B: 15 min to 7%. The phenolic components in the extract were calculated in mg GAE/100 g sample dry base (db).

### 2.12 The analysis of pancakes

In the determination of protein, total salt, and soluble and insoluble dietary fiber amounts of pancakes, 960.52, 925.56, and 991.43 official methods of AOAC were used, respectively. Total sugar analyses were carried out by the ICUMSA GS 4/3‐3 method. Total fat analyses were carried out by ISO 17189/IDF 194.

### 2.13 Sensory analyses of pancakes

Sensory analysis was performed at the Laboratory of Functional Foods at Suleyman Demirel University. The objective of sensory evaluation, which employed 16 semi‐trained participants, was to analyze the control and functional pancake's descriptive profile on a 5‐point scale (1 being the most despised and 5 the most liked). The acceptable threshold was represented by the number three. The panelists assessed the pancakes' visual appeal, odor, texture, taste, and overall acceptability (Incoronato et al., 2021).

### 2.14 Statistical analysis

The initial extraction experiments were carried out in triplicate, and the findings were presented as means accompanied by standard deviations (SD). Statistical analysis of the collected data was detected using SPSS statistical software. To assess the significance of disparities among treatments, variance analysis was employed, and significant variations were pinpointed using Duncan's multiple comparison test. The statistical significance was ascertained at a significance level of 0.05 (alpha value).

## 3 RESULTS AND DISCUSSION

### 3.1 Physicochemical analysis of fresh red cabbage juice

The purpose of this study was to evaluate the MW‐HAD foam mat drying process of red cabbage juices, analyze the impacts of foaming agents and drying process variables on RCJP, and develop functional pancakes using the determined optimum RCJP. Fresh red cabbage press juices were used in the study, and analyses of color, *a*_w_, moisture, SSC, pH, titration acidity, AAC, TAC, TEAC, and TPC were conducted before drying (Table 1). These physicochemical parameters of the red cabbage juice used in the research served as the basis for understanding how these values influenced the subsequent foam drying process.

The results of color, moisture, *a*_w_, SSC, pH, % titration acidity, AAC, TAC, TEAC, and TPC of the red cabbage juices used in the study were correlated with other red cabbage studies in the literature (Ghareaghajlou et al., 2021; Heo & Lee, 2006; Inthuja et al., 2020; Leahu et al., 2018; Mezzetti et al., 2022; Sarkar et al., 2021).

### 3.2 Drying characteristics

Analyzing drying characteristics assists researchers in choosing optimal drying methods, controlling the drying process, and identifying ideal moisture removal conditions. Additionally, a fundamental understanding of drying kinetics and modeling is essential for evaluating and potentially enhancing dryers, whether for improvements or scaling up from lab to pilot size (Delfiya et al., 2022).

**FIGURE 1:**
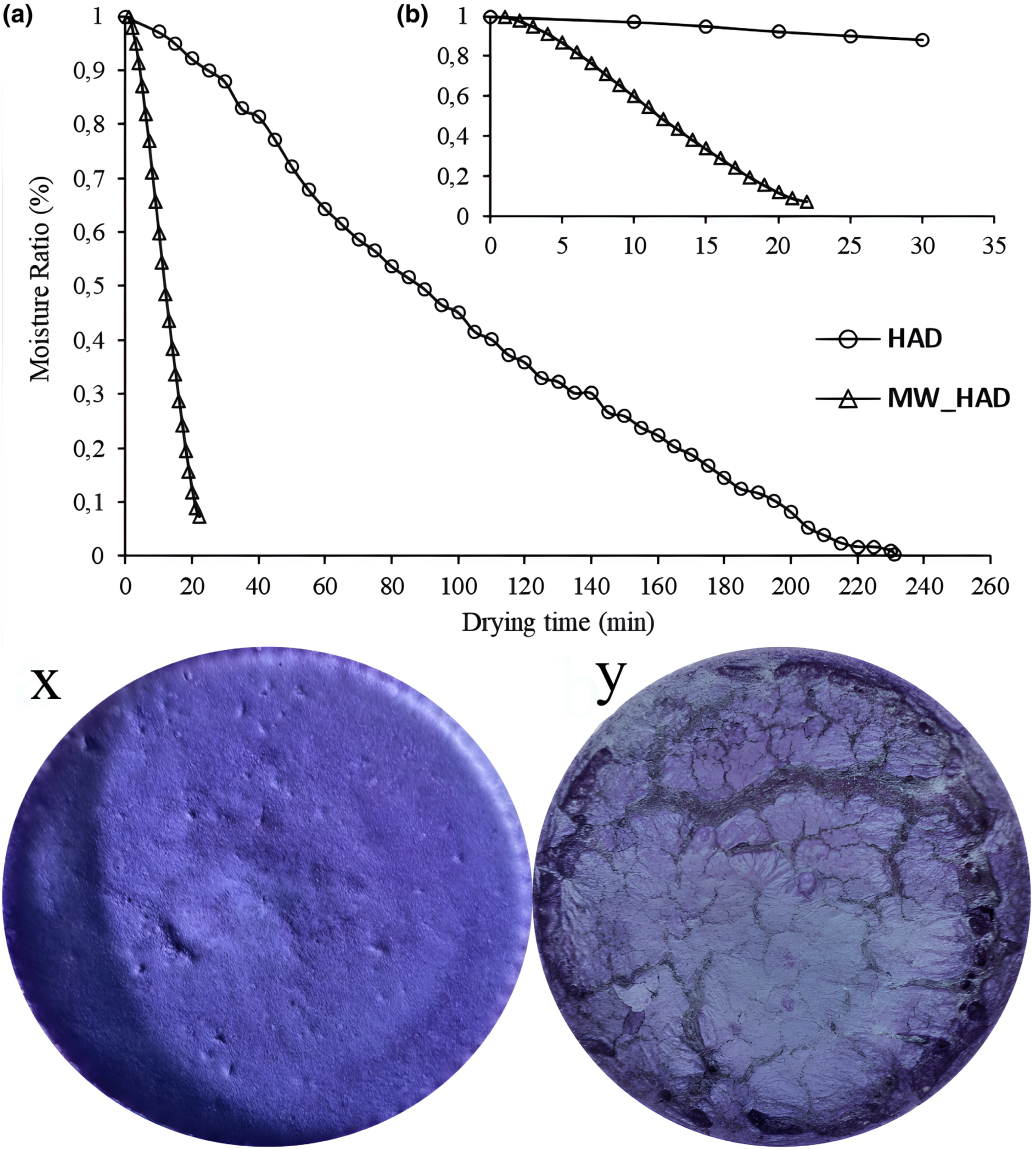
Drying curves of MW‐HAD and HAD: (a) drying time between 0 and 230 min; (b) drying time between 0 and 30; (x) MW‐HAD‐dried sample using optimum parameters; (y) HAD‐dried sample using optimum parameters.

When Figure 1a,b is examined, the drying times of foams created under the same conditions in different drying systems are clearly understood. The drying curve highlights the short drying time achieved with the hybrid MW‐HAD method. The most important difference between hybrid MW‐HAD and HAD systems is the heat transfer mechanisms. While microwave energy affects all layers of the foam, where the water molecule is located, regardless of negative factors such as air bubble insulation, hot air circulation removes the moisture layer accumulated on the drying surface, allowing the foam to heat more equally and quickly. In conventional drying, increasing the surface area for foam drying provides an advantage, but air trapped within the foam acts as an insulator and creates resistance to heat conduction. In addition, it is understood that drying time is very important in foam drying studies when visual x and y are examined. When Figure 1x is examined, MW‐HAD drying was completed in a short time, the foam stability was not impaired, and the structure maintained its integrity during drying. Figure 1y shows that, as a result of the long drying time, the structure lost its integrity, and fractures and cracks occurred in the structure during drying. The instability that occurred in the structure lost the advantage of increasing the drying surface provided by foam drying. In addition, this situation caused some areas to dry out more and some areas to remain more humid, and the drying was not uniform. Shortening the drying time has a positive effect on the physicochemical properties of the product and provides an economic advantage by allowing more products to be dried per unit time. In this context, the MW‐HAD hybrid drying system is more successful than the conventional method.

### 3.3 Determination of physicochemical and functional properties of RCJP with a drying process

In this study, RCJ was subjected to foam‐mat drying utilizing the RSM Box–Behnken experimental design in conjunction with various foaming agents, followed by a MW‐HAD process. Additionally, the physicochemical properties of control (hot air‐drying at 60°C) and optimum (MW‐HAD at 360 W and 60°C) foam‐mat‐dried red cabbage juices were compared. EWP, CMC, and MW power were selected as independent variables. The dependent variables included drying time, color (BI values), TAC, and AAC. The dependent and independent variables used in the optimization are presented in Table 2.

**TABLE 2:** Independent and dependent variables used in optimization: drying time, browning index, total anthocyanin content, and ascorbic acid content of foam‐mat‐dried RCJP.

Run^a^	MW (W)	EWP (%)	CMC (%)	DT	BI	TAC	AAC
1	270	10	1.2	26	10.56	80.41	59.90
2	180	2	0.75	33	9.61	164.03	75.93
3	180	10	0.75	31	16.15	108.88	33.44
4	270	6	0.75	25	14.94	143.19	59.71
5	360	10	0.75	21	19.55	102.99	71.60
6	270	6	0.75	25	15.29	130.91	53.91
7	270	2	0.3	27	22.42	120.71	46.91
8	270	6	0.75	25	23.15	141.52	50.83
9	270	10	0.3	27	16.38	123.33	59.82
10	270	2	1.2	26	36.28	225.77	46.34
11	360	6	0.3	21	42.29	209.32	46.31
12	360	2	0.75	21	73.27	172.95	25.02
13	180	6	1.2	33	17.85	199.21	52.64
14	180	6	0.3	32	16.57	74.72	50.1
15	360	6	1.2	20	40.55	85.80	48.2

Abbreviations: AAC, ascorbic acid content (mg/100 g db); BI, browning index; CMC, carboxymethylcellulose; DT, drying time (min); EWP, egg white protein; MC, moisture content (%); MW, microwave; TAC, total anthocyanin content (mg cyanidin3g/100 g db); WA, water activity (*a*_w_).

^a^ Randomly distributed.

The drying time ranged between 20 and 33 min, and these fluctuations were markedly impacted by diverse drying and foaming parameters, leading to a broad spectrum of drying times throughout the drying procedures (Table 2). To our knowledge, no prior investigations in the scientific literature have explored the process of foam‐mat drying red cabbage juice. However, foam‐mat drying of various vegetables and fruits has been reported (Kumar et al., 2022). Similar to our findings, previous studies have shown reductions in drying times by 25%–90% (Ozcelik et al., 2019; Prabhanjan et al., 1995; Rzepecka et al., 1976). The reduction in drying time during MW‐HAD foam‐mat drying can be attributed to several factors working in synergy. First, the foaming of liquids, aided by the inclusion of EWP and CMC, leads to the creation of microscopic bubbles within the foamed mass. This foaming phenomenon significantly expands the available surface area for moisture evaporation, enhancing mass transfer. Additionally, as noted by previous studies (Aydar et al., 2022; de Cól et al., 2021; Zielinska et al., 2020), increasing the concentration of foaming agents and microwave power has been shown to further reduce the drying time. The reduction in drying time can be explained by the penetration of microwave energy deep into the substance. Microwave heating generates a substantial difference in vapor pressure between the substance's interior and exterior, promoting rapid moisture migration and evaporation. The efficient volumetric heating provided by microwaves, with minimal thermal lag, facilitates this process. As a result, the combined effects of foaming agents and microwave energy contribute to a significant reduction in the overall drying time.

The second dependent variable used in the optimization is the BI, and the values were found to be between 9.61 and 73.27, which were greatly affected by different drying and foaming conditions (Table 2). The observed browning reaction during heat treatment has been expressed in previous literature, with elevated BI values serving as an indicator of more significant browning (Abano et al., 2011; Cernîşev, 2010). Consistent with our findings, research on foam‐mat drying of various fruits and vegetables has shown that BI increases with the power of MW and drying temperature. The concentrations of EWP and CMC also influence BI, leading to either an increase or a decrease (Kumar et al., 2022). Increasing MWP may induce disruption of the structure of foamed RCJ, resulting in the development of a brownish appearance in the RCJP. Similar results were also shown in the literature (Gao et al., 2022; Marzuki et al., 2021). This observed browning can be correlated with pigment degradation, as previously explored by Özkan Karabacak et al. (2023), and the occurrence of the Maillard reaction, as discussed in the study by de Cól et al. (2021).

The amount of anthocyanins as the third dependent variable was significantly influenced by various drying and foaming methods, similar to the other two dependent variables. According to Table 2, the total anthocyanin content ranged from 74.72 to 225.77 mg cyanidin3g/100 g db, demonstrating the impact of different drying and foaming conditions on the content. Red cabbage anthocyanins are highly valued in the food industry as natural coloring agents due to their ability to provide color across a wide pH range compared to other natural anthocyanins (Ghareaghajlou et al., 2022). The amount of anthocyanins in red cabbage is higher than in most other foods and is influenced by factors such as agricultural methods, pesticide use, variety, and the maturation period of red cabbage (Podsedek et al., 2017).

As the last variable considered in the optimization, the ascorbic acid content was highly affected by different drying and foaming conditions. The range of values for AAC obtained was from 25.02 to 75.93 mg/100 g db (Table 2). Vitamin C, important antioxidant with numerous health benefits, is commonly found in a variety of plant‐based foods and should be included in a regular diet for good health. However, vitamin C is susceptible to deterioration during fruit and vegetable preparation, mainly due to heat and air exposure. Consequently, vitamin C is widely used as a marker of overall quality loss during processing and storage. Traditional preservation methods, such as drying or freezing, generally lead to a reduction in vitamin C content (Giannakourou & Taoukis, 2021).

In this context, it is critical to examine the effect of each stage (MW power, EWP, and CMC concentration) and to mathematically define the factors that affect drying time (DT), BI, TAC, and AAC stability in order to improve the drying using innovative techniques such as foam‐mat MW‐HAD.

### 3.4 Model optimization of dependent variables

The objective of the study was to optimize the foam‐mat MW‐HAD drying and foaming process conditions in terms of drying time, BI, moisture, *a*_w_, total anthocyanin, and ascorbic acid contents using RSM. The DT, BI, TAC, and AAC were identified as significant response variables for model optimization. Table 3 shows the model parameters and regression coefficients of the generated models for foam‐mat MW‐HAD drying red cabbage juices. The regression model employed a significance level (alpha value) of 0.05 and used backward elimination regression to progressively eliminate variables and identify the most accurate explanation for the data. The findings revealed that the developed models for DT, BI, TAC, and AAC in the dried samples successfully accounted for the variations observed in the data with respect to the process parameters. As indicated in Table 3, these models demonstrated an explanation of over 90% of the variation in all dependent variables and exhibited statistical significance for these four responses (*p* ≤ .001).

**TABLE 3:** Coefficients and lack‐of‐fit values of the equations derived from the models for drying time, BI, TAC, and AAC of foam‐mat‐dried red cabbage juices.

Variables^a^	DT^b^	BI	TAC	AAC
*β*_0_	53.31*	10.1*	−235.6*	154.3*
*β*_1_ (X_1_)	−0.1130*	−0.230*	1.1818^ns^	−0.400^ns^
*β*_2_ (X_2_)	−1.00^ns^	8.83*	7.04*	−15.74***
*β*_3_ (X_3_)	−2.78^ns^	‐	554.2***	‐
*β*_11_ (X_1_X_1_)	0.000093***	0.001188**	‐	‐
*β*_22_ (X_2_X_2_)	0.0469***	‐	‐	‐
*β*_33_ (X_3_X_3_)	3.70***	‐	‐	‐
*β*_12_ (X_1_X_2_)	0.001389^ns^	−0.04185*	‐	0.06185*
*β*_13_ (X_1_X_3_)	−0.01235^ns^	‐	−1.531*	‐
*β*_23_ (X_2_X_3_)	‐	‐	−20.55*	‐
Model	*	*	*	*
$R_{\mathrm{adj}}^{2}$	98.72	91.00	97.57	90.63
$R_{\text{pred}}^{2}$	93.44	83.90	89.36	84.27
Lack of fit	0.784	0.525	0.354	0.706

^a^ The constant coefficient is denoted as *β*_0_, representing the baseline value. The linear coefficient, *β*_*i*_, reflects the main effect of each variable. The quadratic coefficient, *β*_*ii*_, signifies the presence of a quadratic relationship between the variable and the response. The two‐factor interaction coefficient, *β*_*ij*_, accounts for the combined effect of two variables on the response.

^b^ Capital letter represents the following corresponding names: AAC: ascorbic acid content (mg AA/100 g db), BI: browning index, DT: drying time (h), TAC: total anthocyanin content (mg cyanidin3g/100 g db).

*Significant at *p* ≤ .001; **Significant at *p* ≤ .01; ***Significant at *p* ≤ .05; ^ns^Not significant at *p* > .05. Subscripts X_1_,_2,3,_ represent MW power, EWP, and CMC, respectively.

**FIGURE 2:**
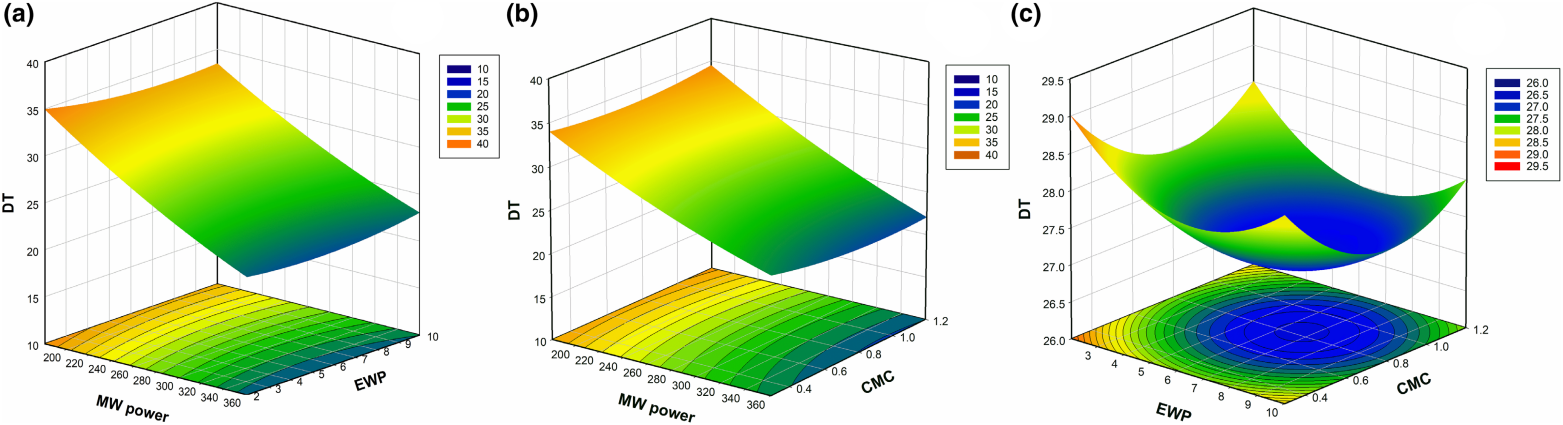
Influence of microwave power (W), EWP (%), and CMC (%) on the drying time (min) of foam‐dried cabbage juice; (a) MW power and EWP, (b) MW power and CMC, (c) EWP and CMC interactions.

The goal of the drying process is to reduce energy consumption while improving the quality of the dried product, so drying time is the most critical independent variable (Motevali et al., 2011). The first model was created for the drying time. After evaluating the mathematical equation, it was found that only one first‐order term, depending on microwave power, had a significant effect. The equation is simplified by removing all non‐significant terms from the quadratic polynomial. Additionally, the lack‐of‐fit test revealed that it was an effective model with no significant fitting issues (*p* > .05). Figure 2a,b illustrate the relationship between drying time and MW power in combination with EWP and CMC, respectively. Notably, Figure 2c highlights the significant curvature effect of EWP and CMC. In significant terms, the curvature effects of EWP and CMC were observed in Figure 2c. According to the current research, it was discovered that increasing the amount of EWP and CMC up to a particular level (5%–7% EWP, 0.6%–0.8% CMC) shortened the drying time while decreasing the drying time at lower and higher levels. Furthermore, an increase in MW power contributed to a shorter drying process duration. This can be attributed to the enhanced transmission of energy to the food material, facilitating increased water evaporation within a given timeframe and thus accelerating the drying process (Putra & Ajiwiguna, 2017). Figure 2a,b demonstrates that the combination of higher EWP levels and lower CMC levels resulted in the shortest drying times. This observation aligns with previous studies on foam‐mat drying that employed EWP and CMC as foaming agents, indicating that these agents aid in the production of low‐moisture powder products (Gao et al., 2022). Although there is no specific study about foam‐dried red cabbage juices, similar findings have been reported for the foam drying of blueberry pulps using MW (Gao et al., 2022). This could be because the inclusion of EWP facilitates the formation of microscopic bubbles in the foamy mass, increasing the surface area for moisture evaporation and therefore accelerating water diffusion and removal (de Cól et al., 2021; Rajkumar et al., 2007). Similar to our results, drying time was reduced by 25%–90% in samples dried using foam‐mat drying techniques for star fruit, pineapple, mango, papaya, banana, corn, red sorghum, and turmeric extract. Modeling studies on MW‐HAD drying cabbages have found that a MW power range of 200–600 W and a HAD temperature range of 60–80°C are the optimum values, which is consistent with our findings (Aghilinategh et al., 2015; Aslan & Ertaş, 2021; Asokapandian et al., 2016; Gupta et al., 2021; Kanha et al., 2022; Qadri et al., 2020; Qadri & Srivastava, 2017).

**FIGURE 3:**
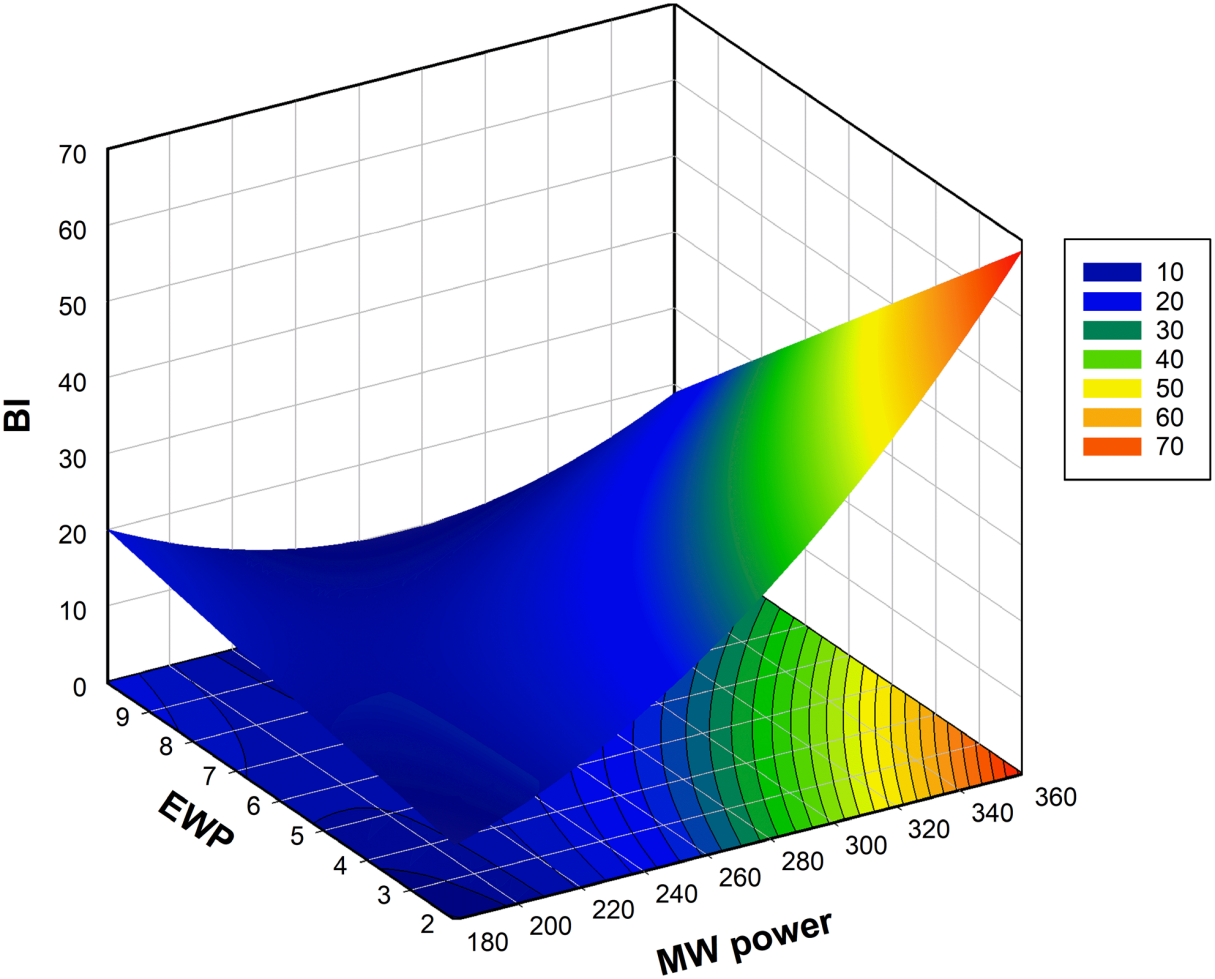
Influence of EWP (%) and microwave power (W) on the browning index of foam‐dried cabbage juice.

The BI was another important dependent variable evaluated in this study, providing insights into the product's degree of heat exposure. In drying experiments, the BI has garnered significant attention in the literature, particularly for red‐colored fruits such as strawberries, as well as fruits like kiwi and bananas (Conti et al., 2014; Crecente‐Campo et al., 2012; Diamante et al., 2010; Nunes & Delgado, 2014; Ornelas‐Paz et al., 2013; Wojdyło et al., 2009). It was discovered that the two first‐order terms, MW power and EWP, had a significant impact when the mathematical equation was examined. Examining the interaction and quadratic relationships, it was discovered that MW power*MW power and MW power*EWP were significant, while the other factors were insignificant. The equation is simplified by removing all non‐significant terms from the quadratic polynomial. Additionally, the lack‐of‐fit test confirmed that the model was effective, with no significant fitting issues (*p* > .05). Figure 3 visually illustrates the changes in the BI. When evaluating the individual impacts of EWP and MW power on the BI, we observed a continuous increase in BI with increasing MW power, accompanied by a slight and limited increase with increasing EWP. When the EWP and MW power effects are considered together, the lowest BI value is obtained at 360 W and 10% EWP. CMC, on the other hand, did not have a statistically significant influence on BI. Our findings align with previous studies on foam‐mat drying of different fruits and vegetables, which reported that higher microwave power or drying temperatures resulted in increased color changes and browning (Gao et al., 2022; Kumar et al., 2022; Qadri, 2022). Additionally, it has been reported that the presence of EWP can expedite the browning reaction in the final drying stage when the sample surface temperature surpasses 100°C. Moreover, the addition of CMC leads to a rise in BI, followed by a decline, as per reports (Gao et al., 2022).

**FIGURE 4:**
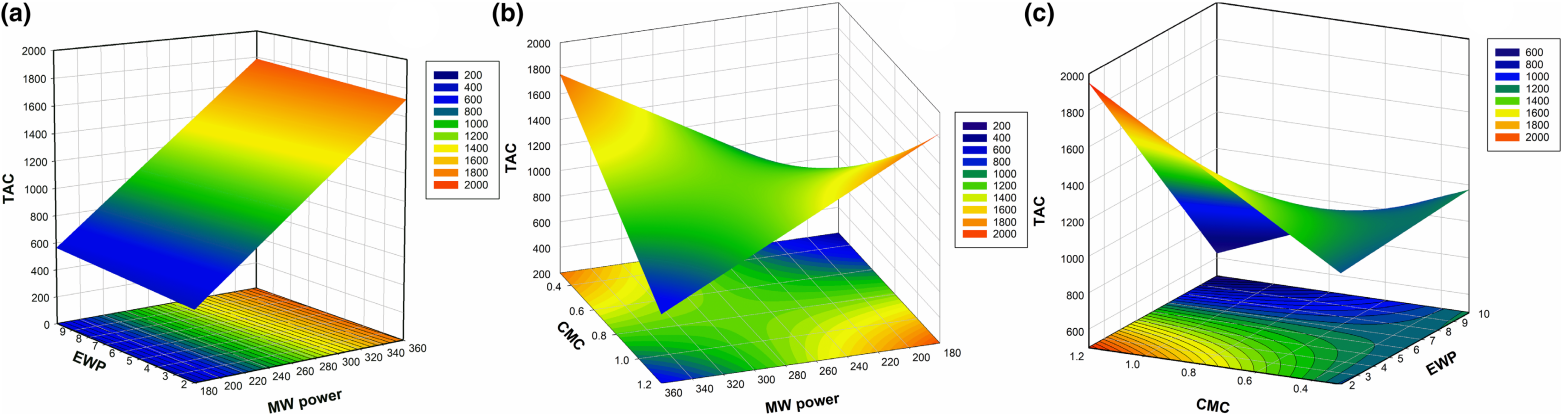
Influence of EWP (%), microwave power (W), and CMC (%) on TAC (mg cyanidin3g/100 g db) of foam‐dried cabbage juice; (a) MW power and EWP, (b) MW power and CMC, (c) EWP and CMC interactions.

Anthocyanin levels were the third dependent variable and showed a significant change with different drying and foaming conditions. Following an analysis of the mathematical equation, it was found that microwave power, which is only a first‐order term, had no impact. Additionally, the equation is simplified by removing all second‐order terms from the model. MW power*CMC and EWP*CMC were shown to be significant interaction terms. Furthermore, the insignificant lack of fit demonstrated that it was an effective model (*p* ≤ .05). Analysis of Figure 4a illustrates that the amount of anthocyanin content does not vary depending on the EWP, but it generally increases linearly as the MW power increases. Consistent with our findings, pathway analysis and degradation kinetic analysis conducted on fruit puree during microwave‐aided foam mat drying revealed that anthocyanin degradation primarily occurs during the final drying process (Sun et al., 2020). Therefore, the use of higher MW power (360 W) may be effective in preserving anthocyanins while achieving faster drying. Figure 4b shows the curvature effect of CMC and MW power on TAC. It is observed that the TAC decreases linearly with increasing CMC and MW values. However, when their interactions are considered, it is understood that the highest TAC is obtained with a low CMC amount and a high MW power. Our findings align with a study on foam‐dried blueberry puree, which reported that increasing the CMC concentration from 0 to 0.5 g/100 g led to an increase in TAC, while further increasing CMC to 1 g/100 g resulted in a decrease (Gao et al., 2022). Similarly, another study on the foam mat drying of mango found that the addition of 0.3 g/100 g CMC increased TAC (Lobo et al., 2017). Similarly, Abbasi and Azizpour (2016) discovered that higher CMC concentrations (1–1.5 g/100 g) drastically decreased TAC.

**FIGURE 5:**
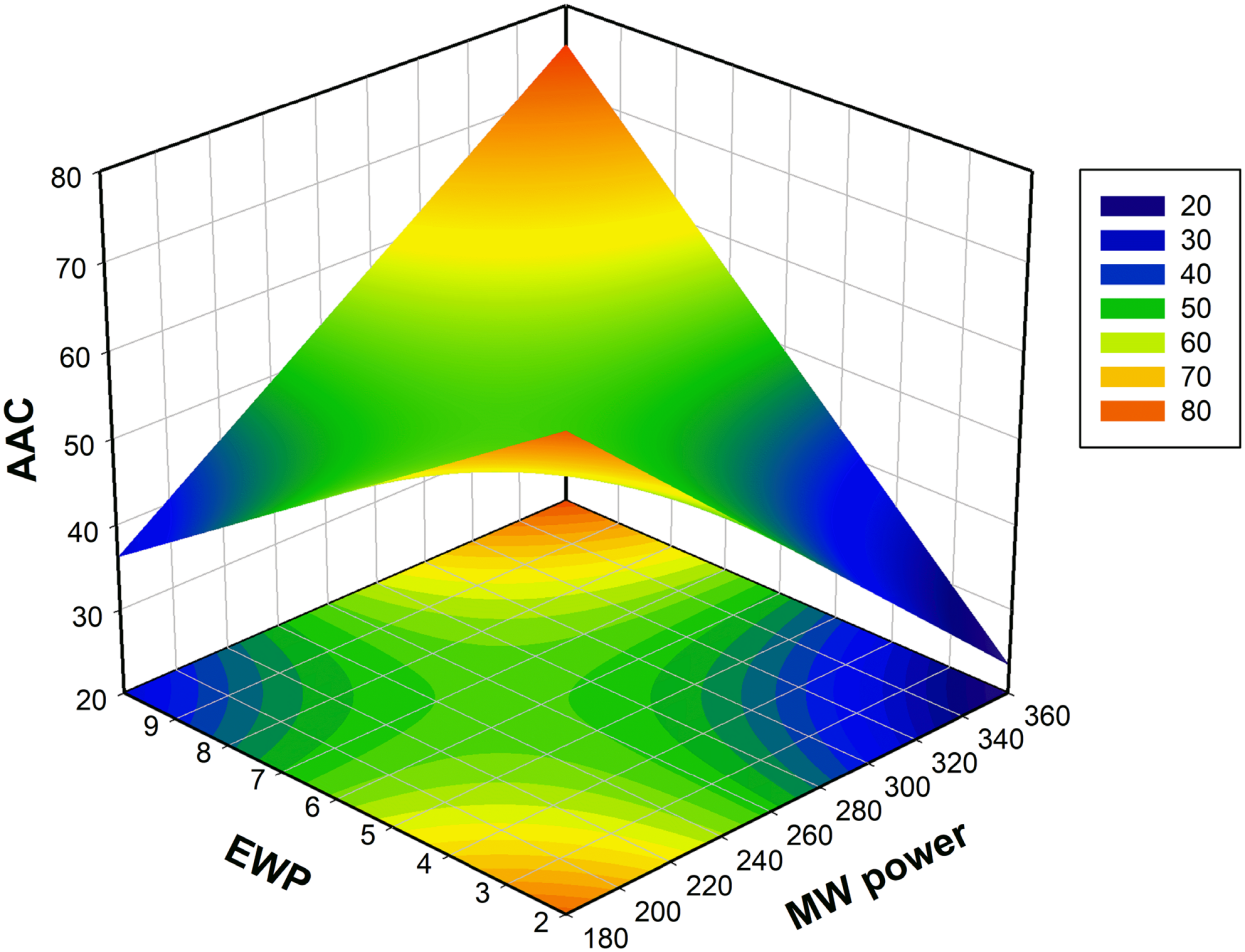
Influence of EWP (%) and microwave power (W) on AAC (mg /100 g db) of foam‐dried cabbage juice.

The fourth dependent model variable was selected as AAC. The mathematical equation became evident that only the first‐order term (EWP) and the second‐order term (EWP*MW power) exerted a noteworthy influence. The equation is simplified in this context by deleting all insignificant terms from the model. In addition, there is no lack of fit problem (*p* ≤ .05). Figure 5 was generated todisplay the impacts of EWP and MW power on ascorbic acid. As EWP and MW power increase linearly, the amount of ascorbic acid decreases. However, when considering the effects of these two variables together, the highest ascorbic acid value is achieved at the highest values of MW power and EWP. In line with our results, a study indicates that increasing the power of the microwave and reducing the thickness of foam can result in a higher content of ascorbic acid in guava fruit dried by foam drying (Qadri & Srivastava, 2017). Another study on foam‐dried tomatoes demonstrated that higher concentrations of EWP resulted in a decrease in ascorbic acid content, aligning with our research findings (Hossain et al., 2021). Furthermore, in a published foam drying study, it was reported that using proteins and agents such as maltodextrin to enhance foam stability instead of CMC proved to be effective in preserving the amount of anthocyanins and ascorbic acid while reducing drying time (Ozcelik et al., 2020).

### 3.5 Optimization and validation of foam‐mat MW‐HAD drying and foaming process conditions in terms of DT, BI, TAC, and AAC

In this study, the desirability function was employed to determine the optimal conditions. The desirability value, ranging from 0 to 1, was calculated using Minitab. A value of 1 indicates the achievement of an ideal outcome, while a value of 0 suggests that one or more responses exceeded the desired limits (Cojocaru et al., 2009). The collected data were analyzed statistically, identifying the dependent variables with high predictive power, namely DT, BI, TAC, and AAC. During the optimization process, TAC and AAC were maximized, while drying time and BI were minimized. Consequently, the optimal values for MW power, EWP, and CMC content were determined to be 360 W, 60°C, 10 g/100 g, and 0.3 g/100 g, respectively. The desirability score obtained was 0.87. To assess the validity of the model and its ability to predict real data, the drying process was carried out under optimized conditions. The model estimated the values of DT, TAC, AAC, and BI for the validation treatments, ranging from 20.7 to 24.3 min, 170.69 to 228.46 mg/100 g dry matter, 64.64 to 86.37 mg/g dry matter, and 4.7 to 33.3, respectively. Statistical analysis of the results demonstrated that the theoretical and experimental data decreased within the confidence intervals for DT and BI, providing effective validation. In addition, the AAC and TAC values were slightly higher than the predicted values. Because of the goals of the experiment, it should be noted that experimental trials were conducted at levels exceeding the predetermined limits for EWP and CMC (12% EWP and 0.2% CMC). These trials were referred to as experiments outside the trial plan (OTP), and the drying analysis results for these values are provided in Table S1. Experimental plans in optimization, EWP and CMC, were 10% and 0.3%, respectively, and trials were performed at levels over these limitations (12% EWP and 0.2% CMC). This was named as the experiment outside the trial plan (OTP), and the drying analysis results at these values are included in Table S1. While the drying time did not change as a result of the OTP experiment, there were statistically significant declines in major chemical analyses such as TAC, AAC, TPC, and TEAC compared to the optimum point, yet browning was determined to be less than the optimum point. The correctness of the chosen optimum point has been verified in this context. The physicochemical parameters of optimum and control RCJP are shown in Table 4.

**TABLE 4:** Physicochemical properties of optimum and control RCJP.

Analyze/Sample	Optimum RCJP	Control RCJP
DR	0.0432	0.0212
FT	0.72 ± 0.01^a^	0.71 ± 0.01^a^
FC	189.53 ± 0.85^a^	188.67 ± 0.21^a^
FS	104.47 ± 0.33^a^	104.13 ± 0.31^a^
BD	0.76 ± 0.01^a^	0.75 ± 0.00^a^
CI	42 ± 0.03^a^	41 ± 0.02^a^
HR	1.72 ± 0.01^a^	1.70 ± 0.01^a^
WAI	3.79 ± 0.01^a^	1.61 ± 0.01^b^
WSI	0.3 ± 0.00^a^	0.6 ± 0.00^b^
SP	5.53 ± 0.03^a^	4.25 ± 0.01^b^
SSC	48.20 ± 0.01^a^	52.66 ± 0.02^b^
MC	8.62 ± 0.36^a^	7.11 ± 0.29^b^
WA	0.34 ± 0.01^a^	0.31 ± 0.01^b^
pH	6.31 ± 0.01^a^	6.34 ± 0.02^a^
DT	21 ± 0.50^a^	231 ± 5.00^b^
BI	12.62 ± 0.75^a^	1.16 ± 0.05^b^
TAC	347.40 ± 4.47_a_	217.80 ± 2.85^b^
AAC	149.6 ± 2.27^a^	62.3 ± 0.55^b^
TEAC	244.53 ± 1.24^a^	161.30 ± 0.24^b^
TPC	371.39 ± 0.41^a^	223.66 ± 0.33^b^

*Note*: The statistical difference between samples is shown by ^a,b^ changes in the same line (*p* ≤ .05).

Abbreviations: AAC, ascorbic acid content (mg/100 g db); BD, bulk density (%); BI, browning index; CI, Carr index (%); DR, Drying rate (average); DT, drying time (min); FC, foam capacity (%); FS, foam stability (%); FT, foam thickness in tray (mm); HR, Hausner ratio; MC, moisture content (%); OTP, Outside the trial plan (EWP 12 g/100 g, CMC 0.2 g/100 g, 360 W‐60°C); SP, swelling power (%); SSC, total soluble solid content; TAC, total anthocyanin content (mg cyanidin3g/100 g db); TEAC, trolox equivalent antioxidant capacity (mg/100 g db); TPC, total phenolic content (mg GAE/100 g db); WA, water activity (*a*_w_); WAI, water absorption index (%); WSI, water solubility index (%).

Compared to HAD, the average drying rate is 50.92% higher in samples dried with MW‐HAD. The decrease in drying time during the MW‐HAD foam‐mat drying process results from foaming, which significantly expands the surface area and enhances mass transfer. Additionally, microwave heating offers immediate, volumetric heating without any thermal delay. The drying rate in MW‐HAD drying processes was calculated to be greater than in traditional drying, which is consistent with our findings (Kumar et al., 2022; Reis et al., 2021).

While foam volume and stability provide critical information about the behavior and properties of the foam, mass density, Hausner ratio, and Carr index allow the flow behavior of powder products to be evaluated based on mass densities. In this context, the produced powders can be classified as materials with low fluidity (Hausner ratio >1.2–1.4 and Carr index >18%–25%) (Szulc & Lenart, 2016). When comparing the FT, FC, FS, BD, CI, and HR values of the control and optimum samples using the same volume of cabbage juice, no statistically significant difference was observed. WAI, WSI, SP, and SSC values were statistically different in two different drying systems, and optimum RCJP samples had higher WAI and SP values. However, WSI and SSC values were higher in the control RCJP. In parallel with our findings, it was reported that the water holding capacity and water solubility decreased with the increase of MW power in yogurt and fig samples that were foam‐dried with a microwave (Varhan et al., 2019; Yüksel, 2021). While there was no difference in pH values between the two drying techniques, the MC and WA values were significantly higher. It was reported, similarly to our findings in the present research, that MC and WA values were higher in foam drying with MW compared to convection drying due to non‐uniform heating (Kumar et al., 2022). When comparing the drying time (DT), it was found that the drying time of the optimum RCJP was 90.9% lower than that of the control RCJP samples (Table 4). In this context, the combination of MW and hot air provides volumetric rapid heating as well as speeds up moisture removal from the matrix by sweeping wet air from the environment thanks to air circulation. However, this situation has a negative effect on BI. The BI values in the optimum samples produced by MW drying were found to be significantly higher than in the control samples. This can be observed in circumstances where some burning occurs as a result of the fast evaporation of moisture on the structure's surface at high watt values due to volumetric and rapid heating in MW drying. Similar results on foam drying with MW have been published in the literature (Gao et al., 2022; Kumar et al., 2022; Qadri, 2022). The optimum drying rates of RCJP, produced via MW‐HAD drying, were substantially greater for TAC, AAC, TEAC, and TPC than the control. Similar results have been found in the literature, arguing that this is due to the fact that the materials dried with MW‐HAD dry out in a relatively short time and that these values decrease with increasing drying time (Joudi‐Sarighayeh et al., 2022; Kumar et al., 2022; Qadri & Srivastava, 2017; Sun et al., 2020).

### 3.6 Development of functional pancakes and comparison of their physical, chemical, and sensory properties to control pancakes

Consumers place a high priority on the nutritive content and sensory qualities of the products they consume. Pancakes were prepared as given under method 2.2.3. Pancakes consisted of wheat flour, eggs, salt, sugar, milk, and water. In the formulation of functional pancakes, unlike control pancakes, 20 g of RCJP obtained with MW‐HAD at optimum conditions was added instead of wheat flour. The parameters of the functional and control pancake samples' physicochemical properties, such as moisture, *a*_w_, color, pH, TAC, AAC, TEAC, and TPC, as well as their protein, dietary fiber, salt, sugar, and fat contents, are given in Table 5.

**TABLE 5:** Physicochemical properties of functional and control pancakes.

Analyze/Sample	Functional pancakes	Control pancakes
MC	53.54 ± 1.18^a^	47.46 ± 1.31^b^
WA	0.737 ± 0.00^a^	0.711 ± 0.01^b^
L*	65.01 ± 1.97^a^	53.87 ± 1.28^b^
a*	12.09 ± 1.42^a^	13.14 ± 1.09^a^
b*	31.32 ± 0.29^a^	27.68 ± 0.20^b^
BI	76.75 ± 1.02^a^	86.80 ± 1.11^b^
TAC	28.64 ± 0.08	‐
AAC	18.7 ± 0.01	‐
TEAC	326.39 ± 2.84^a^	131.99 ± 0.37^b^
TPC	130.07 ± 4.21^a^	31.37 ± 1.21^b^
Protein (%)	15.31 ± 0.16^a^	9.94 ± 0.08^b^
Total salt (%)	1.87 ± 0.01^a^	1.64 ± 0.01^b^
Total sugar (%)	228.07 ± 0.19^a^	102.82 ± 0.52^b^
Total fat (%)	2.56 ± 0.11^a^	3.11 ± 0.08^b^
Total dietary fiber (%)	8.69 ± 0.21^a^	1.48 ± 0.03^b^

*Note*: The statistical difference between samples is shown by ^a,b^ changes in the same line (*p* ≤ .05).

Abbreviations: AAC, ascorbic acid content (mg/100 g db); BI, browning index; *L*, *a*, *b*, *C*, h°, color values; MC, moisture content (%); TAC, total anthocyanin content (mg cyanidin3g/100 g db); TEAC, μmol T.E/100 g wb; TPC, total phenolic compound mg GAE/100 g db; WA, water activity (*a*_w_).

The RCJP used to make functional pancakes has a moisture content under 10%. This demonstrated that it would not affect the flour's quality during storage or accelerate the growth of microbes, insect infestation, or aggregation (Aziah & Komathi, 2009; Leão et al., 2017; Mashau et al., 2020). When the moisture and water activity values of pancakes made with the same baking time and total dough weight are compared, functional pancakes made with RCJP have a higher content. This could be due to the higher water retention in the structure of RCJP produced with MW‐HAD, which is utilized to make functional pancakes (Table 4). When considering the color values of both pancakes, functional pancakes exhibit higher L* and b* values while demonstrating lower a* values. (Table 5). These numbers show that functional pancakes are less brown, more yellow, and lighter in color than the control. In this situation, the BI value, also known as a measure of exposure to heat treatment, was discovered to be 13.16% lower in functional pancakes and revealed results that were identical to the color values. The analytical results of TAC and AAC are critical, as they were not detectable in control pancakes. Considering the high levels of anthocyanin and ascorbic acid content found in RCJP (Table 4), pancakes made with RCJP retained these beneficial components despite the heat treatment during cooking. The results revealed that the functional pancakes included more polyphenolic compounds and had a higher TEAC value than the control. The protein, carbohydrate, and dietary fiber content of functional pancakes were 35%, 54.91%, and 82.97% higher due to egg white protein and CMC in RCJP (Table 5). While functional pancakes had a higher total salt level than the control, they had a lower total fat content. Red cabbage sodium, potassium, and chlorine components raised the salt content of functional pancakes marginally, but this increase was not statistically significant. The statistical analysis revealed a significantly lower overall fat content, while the functional product exhibited a low‐fat content along with high levels of protein, dietary fiber, phenolic compounds, antioxidants, and anthocyanins. As a result, RCJP could attract the interest of both consumers and producers, as well as promote their valorization in accordance with the functional food approach's purpose.

**FIGURE 6:**
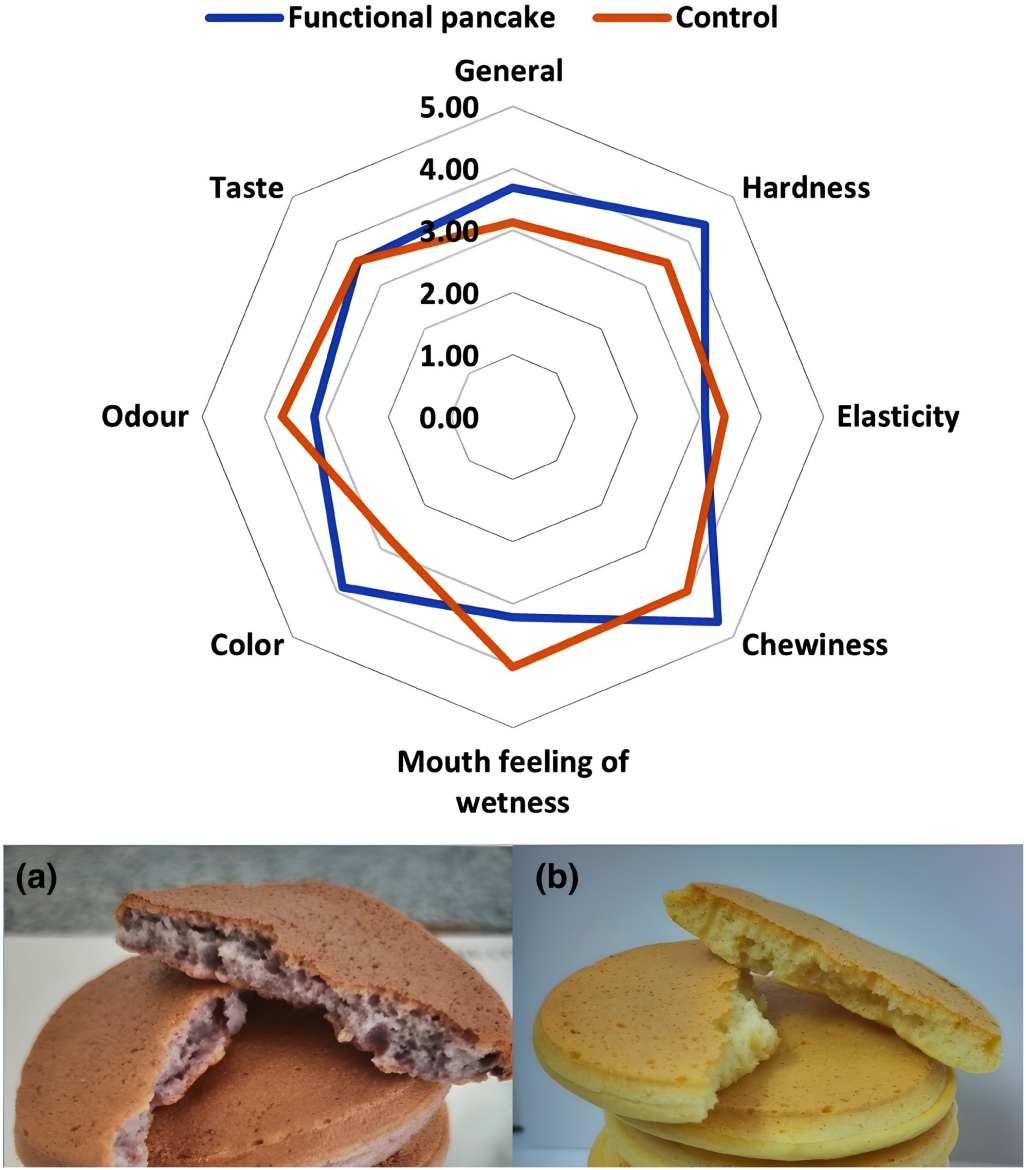
Sensory evaluation results and visuals of pancakes, (a) using RCJP, (b) control.

Consumer behavior related to food selection is known to be influenced not only by price, nutritional content, and health advantages but also by sensory evaluation of the food product. The desired quality parameters of pancakes are not limited to taste and good odor; their soft and porous structure also contributes to their attractiveness. Figure 6 shows the differences in sensory qualities of pancakes produced with RCJP and with flour as a control. The optimized formulation for pancake production was achieved through the preliminary sensory analysis of pancakes. Sensory analyses of functional and control pancakes are given in Figure 6.

When the sensory qualities of pancakes produced with RCJP and wheat flour were assessed, it was found that there were significant differences in chewiness properties in addition to surface color and hardness (*p* ≤ .05), but no significant differences in taste (*p* > .05). The external color of the functional pancakes did not differ greatly between those prepared with RCJP and those created with flour, but the interior of the RCJP pancakes was a pleasing purple color (Figure 6). Aside from its appealing color, it will also make sure that individuals who dislike or consume less red cabbage in their daily routines will still benefit from its therapeutic ingredients, such as anthocyanins, in this way.

According to the results of sensory analysis, elasticity and wetness in the mouth values were felt more in the control samples, but the moisture and *a*_w_ values of the control samples were lower than the pancakes made using RCJP. Although pancakes made with RCJP contained more moisture, it was understood that the structure held tighter than the control samples. The unique herb smell of fresh cabbage was not perceived by the panelists in the pancakes. This can be considered as a positive feature for those who do not like the cabbage smell of RCJP. Finally, when comparing functional pancakes prepared with RCJP substitution to control pancakes prepared with gluten‐free flour in terms of overall appreciation, it was observed that functional pancakes received higher ratings. However, statistical analysis revealed that the difference in appreciation between the two types of pancakes was not statistically significant (*p* > .05).

## 4 CONCLUSIONS

Fruits and vegetables with a high water content, which are also sensitive to heat, sticky, and contain peels and seeds that are therefore difficult to dry using traditional methods, can be dried quickly using the MW‐HAD foam‐mat technique while protecting their nutritional qualities. The potential of foam‐mat drying for red cabbage, which has a high economic value and is particularly rich in anthocyanins, was investigated in the current study. The foam‐mat drying process was modeled and adjusted to ensure maximum quality and performance. Models with high prediction performance were utilized in the optimization. As optimal conditions, the drying MW‐HAD power and temperature, EWP, and CMC concentrations were found to be 360 W‐60°C, 10 g/100 g, and 0.3 g/100 g respectively. The results for DT, BI, TAC, and AAC were satisfied; thus, foam mat drying using MW‐HAD has been found to be an effective method for drying foods with high water content and high bioactive components, such as red cabbage. Compared with the control, a positive effect of shorter drying was observed on almost all quality parameters, e.g. TAC, TPC, AAC, and higher antioxidant activity. For the first time, this study examined the production of foam‐mat‐dried red cabbage powder and its use in pancake production. Pancakes made with MW‐HAD RCJP have a better nutritional value than control pancakes (35% protein, 76% TPC, and 83% dietary fiber) and also include anthocyanins. In this context, the results indicate that MW‐HAD foam mat‐dried RCJP can improve the nutritional and sensory quality of pancakes. Finally, pancakes produced from RCJP were found to be successful with their functional features, attractive color, and superior sensory features.

## AUTHOR CONTRIBUTIONS

**Muhammed Mustafa Ozcelik:** Formal analysis (equal); investigation (equal); methodology (equal); writing – original draft (lead). **Sedef Aydin:** Formal analysis (equal); investigation (equal); methodology (equal). **Ebru Aydin:** Formal analysis (equal); investigation (equal); methodology (equal); writing – review and editing (equal). **Gulcan Ozkan:** Investigation (equal); methodology (equal); supervision (equal); writing – review and editing (equal).

## FUNDING INFORMATION

This research was funded by the Suleyman Demirel University Scientific Research Projects Unit, project number FBY‐2018‐6027.

## CONFLICT OF INTEREST STATEMENT

The authors declare no conflicts of interest.

## Supporting information


Table S1

## Data Availability

The data that support the findings of this study are available on request from the corresponding author.

## References

[fsn33847-bib-0001] Abano, E. E. , Ma, H. , & Qu, W. (2011). Influence of air temperature on the drying kinetics and quality of tomato slices. Journal of Food Processing & Technology, 2, 2–9.

[fsn33847-bib-0002] Abbasi, E. , & Azizpour, M. (2016). Evaluation of physicochemical properties of foam mat dried sour cherry powder. LWT ‐ Food Science and Technology, 68, 105–110.

[fsn33847-bib-0003] Abbaspour‐Gilandeh, Y. , Kaveh, M. , Fatemi, H. , & Aziz, M. (2021). Combined hot air, microwave, and infrared drying of hawthorn fruit: Effects of ultrasonic pretreatment on drying time, energy, qualitative, and bioactive compounds' properties. Food, 10, 1006. 10.3390/foods10051006 PMC8147953 34064476

[fsn33847-bib-0004] Aghilinategh, N. , Rafiee, S. , Hosseinpour, S. , Omid, M. , & Mohtasebi, S. S. (2015). Optimization of intermittent microwave–convective drying using response surface methodology. Food Science & Nutrition, 3, 331–341. 26286706 10.1002/fsn3.224 PMC4534160

[fsn33847-bib-0005] Ahmad, M. , Baba, W. N. , Wani, T. A. , Gani, A. , Gani, A. , Shah, U. , Wani, S. M. , & Masoodi, F. A. (2015). Effect of green tea powder on thermal, rheological & functional properties of wheat flour and physical, nutraceutical & sensory analysis of cookies. Journal of Food Science and Technology, 52, 5799–5807. 26344994 10.1007/s13197-014-1701-3 PMC4554617

[fsn33847-bib-0006] Ai, Z. , Ren, H. , Lin, Y. , Sun, W. , Yang, Z. , Zhang, Y. , Zhang, H. , Yang, Z. , Pandiselvam, R. , & Liu, Y. (2022). Improving drying efficiency and product quality of *Stevia rebaudiana* leaves using innovative medium‐and short‐wave infrared drying (MSWID). Innovative Food Science and Emerging Technologies, 81, 103154.

[fsn33847-bib-0007] Aslan, M. , & Ertaş, N. (2021). Foam drying of aquafaba: Optimization with mixture design. Journal of Food Processing and Preservation, 45, e15185.

[fsn33847-bib-0008] Asokapandian, S. , Venkatachalam, S. , Swamy, G. J. , & Kuppusamy, K. (2016). Optimization of foaming properties and foam mat drying of muskmelon using soy protein. Journal of Food Process Engineering, 39, 692–701.

[fsn33847-bib-0009] Ávila, S. , Zalamanski, S. , Tanikawa, L. M. , Kruger, C. C. H. , & Ferreira, S. M. R. (2023). Influence of cooking methods on in vitro bioaccessibility of phenolics, flavonoids, and antioxidant activity of red cabbage. Plant Foods for Human Nutrition, 78, 124–131. 36357658 10.1007/s11130-022-01027-5

[fsn33847-bib-0010] Aydar, A. Y. , Aydın, T. , Yılmaz, T. , Kothakota, A. , Terezia, S. C. , Leontin, C. F. , & Pandiselvam, R. (2022). Investigation on the influence of ultrasonic pretreatment on color, quality and antioxidant attributes of microwave dried *Inula viscosa* (L.). Ultrasonics Sonochemistry, 90, 106184. 36194948 10.1016/j.ultsonch.2022.106184 PMC9531285

[fsn33847-bib-0011] Aydin, E. , Turgut, S. S. , Aydin, S. , Cevik, S. , Ozcelik, A. , Aksu, M. , Ozcelik, M. M. , & Ozkan, G. (2023). A new approach for the development and optimization of gluten‐free noodles using flours from byproducts of cold‐pressed okra and pumpkin seeds. Food, 12, 2018. 10.3390/foods12102018 PMC10216911 37238836

[fsn33847-bib-0012] Aziah, A. A. N. , & Komathi, C. A. (2009). Physicochemical and functional properties of peeled and unpeeled pumpkin flour. Journal of Food Science, 74, S328–S333. 19895499 10.1111/j.1750-3841.2009.01298.x

[fsn33847-bib-0013] Bagheri, H. (2020). Application of infrared heating for roasting nuts. Journal of Food Quality, 2020, 1–10.

[fsn33847-bib-0014] Buko, V. , Zavodnik, I. , Kanuka, O. , Belonovskaya, E. , Naruta, E. , Lukivskaya, O. , Kirko, S. , Budryn, G. , Żyżelewicz, D. , & Oracz, J. (2018). Antidiabetic effects and erythrocyte stabilization by red cabbage extract in streptozotocin‐treated rats. Food & Function, 9, 1850–1863. 29517782 10.1039/c7fo01823a

[fsn33847-bib-0083] Çalışkan Koç, G., Tekgül, Y., Yüksel, A. N., Khanashyam, A. C., Kothakota, A., & Pandiselvam, R. (2022). Recent development in foam‐mat drying process: Influence of foaming agents and foam properties on powder properties. Journal of Surfactants and Detergents, 25(5), 539–557. Portico. https://doi.org/10.1002/jsde.12608

[fsn33847-bib-0015] Cernîşev, S. (2010). Effects of conventional and multistage drying processing on non‐enzymatic browning in tomato. Journal of Food Engineering, 96, 114–118.

[fsn33847-bib-0016] Chauhan, E. S. , Tiwari, A. , & Singh, A. (2016). Phytochemical screening of red cabbage (*Brassica oleracea*) powder and juice‐A comparative study. Journal of Medicinal Plants Studies, 4, 196–199.

[fsn33847-bib-0017] Chelladurai, S. J. S. , Murugan, K. , Ray, A. P. , Upadhyaya, M. , Narasimharaj, V. , & Gnanasekaran, S. (2020). Optimization of process parameters using response surface methodology: A review. Materials Today Proceedings, 37, 1301–1304.

[fsn33847-bib-0018] Cojocaru, C. , Khayet, M. , Zakrzewska‐Trznadel, G. , & Jaworska, A. (2009). Modeling and multi‐response optimization of pervaporation of organic aqueous solutions using desirability function approach. Journal of Hazardous Materials, 167, 52–63. 19179007 10.1016/j.jhazmat.2008.12.078

[fsn33847-bib-0019] Conti, S. , Villari, G. , Faugno, S. , Melchionna, G. , Somma, S. , & Caruso, G. (2014). Effects of organic vs. conventional farming system on yield and quality of strawberry grown as an annual or biennial crop in southern Italy. Scientia Horticulturae, 180, 63–71.

[fsn33847-bib-0020] Crecente‐Campo, J. , Nunes‐Damaceno, M. , Romero‐Rodríguez, M. A. , & Vázquez‐Odériz, M. L. (2012). Color, anthocyanin pigment, ascorbic acid and total phenolic compound determination in organic versus conventional strawberries (*Fragaria×ananassa* Duch, cv Selva). Journal of Food Composition and Analysis, 28, 23–30.

[fsn33847-bib-0021] de Cól, C. D. , Tischer, B. , Hickmann Flôres, S. , & Rech, R. (2021). Foam‐mat drying of bacaba (*Oenocarpus bacaba*): Process characterization, physicochemical properties, and antioxidant activity. Food and Bioproducts Processing, 126, 23–31.

[fsn33847-bib-0022] de la Hera, E. , Gomez, M. , & Rosell, C. M. (2013). Particle size distribution of rice flour affecting the starch enzymatic hydrolysis and hydration properties. Carbohydrate Polymers, 98, 421–427. 23987363 10.1016/j.carbpol.2013.06.002

[fsn33847-bib-0023] de Souza, A. U. , Corrêa, J. L. G. , Tanikawa, D. H. , Abrahão, F. R. , de Jesus Junqueira, J. R. , & Jiménez, E. C. (2022). Hybrid microwave‐hot air drying of the osmotically treated carrots. LWT ‐ Food Science and Technology, 156, 113046.

[fsn33847-bib-0024] Delfiya, D. S. A. , Prashob, K. , Murali, S. , Alfiya, P. V. , Samuel, M. P. , & Pandiselvam, R. (2022). Drying kinetics of food materials in infrared radiation drying: A review. Journal of Food Process Engineering, 45, e13810.

[fsn33847-bib-0025] Diamante, L. , Durand, M. , Savage, G. , & Vanhanen, L. (2010). Effect of temperature on the drying characteristics, colour and ascorbic acid content of green and gold kiwifruits. International Food Research Journal, 45, 441–451.

[fsn33847-bib-0026] Drozdowska, M. , Leszczyńska, T. , Koronowicz, A. , Piasna‐Słupecka, E. , Domagała, D. , & Kusznierewicz, B. (2020). Young shoots of red cabbage are a better source of selected nutrients and glucosinolates in comparison to the vegetable at full maturity. European Food Research and Technology, 246, 2505–2515.

[fsn33847-bib-0027] Gao, R. , Xue, L. , Zhang, Y. , Liu, Y. , Shen, L. , & Zheng, X. (2022). Production of blueberry pulp powder by microwave‐assisted foam‐mat drying: Effects of formulations of foaming agents on drying characteristics and physicochemical properties. LWT ‐ Food Science and Technology, 154, 112811.

[fsn33847-bib-0028] Ghareaghajlou, N. , Hallaj‐Nezhadi, S. , & Ghasempour, Z. (2021). Red cabbage anthocyanins: Stability, extraction, biological activities and applications in food systems. Food Chemistry, 365, 130482. 34243124 10.1016/j.foodchem.2021.130482

[fsn33847-bib-0029] Ghareaghajlou, N. , Hallaj‐Nezhadi, S. , & Ghasempour, Z. (2022). Nano‐liposomal system based on lyophilization of monophase solution technique for encapsulating anthocyanin‐rich extract from red cabbage. Dyes and Pigments, 202, 110263.

[fsn33847-bib-0030] Giannakourou, M. C. , & Taoukis, P. S. (2021). Effect of alternative preservation steps and storage on vitamin C stability in fruit and vegetable products: Critical review and kinetic modelling approaches. Food, 10, 2630. 10.3390/foods10112630 PMC8619176 34828909

[fsn33847-bib-0031] Goyal, A. , Sharma, V. , Sihag, M. K. , Tomar, S. K. , Arora, S. , Sabikhi, L. , & Singh, A. K. (2015). Development and physico‐chemical characterization of microencapsulated flaxseed oil powder: A functional ingredient for omega‐3 fortification. Powder Technology, 286, 527–537.

[fsn33847-bib-0032] Gupta, V. , Prabhakar, P. K. , Gharde, S. , Nimbaria, A. , Sharma, V. , & Rawat, A. (2021). Foam mat drying of jujube (*Ziziphus mauritiana*) juice: Process optimisation, Physico‐functional, phenolic content and antioxidant analysis. Journal of The Institution of Engineers (India): Series A, 102, 1013–1025.

[fsn33847-bib-0033] Heo, H. J. , & Lee, C. Y. (2006). Phenolic phytochemicals in cabbage inhibit amyloid β protein‐induced neurotoxicity. LWT ‐ Food Science and Technology, 39, 331–337.

[fsn33847-bib-0034] Hnin, K. K. , Zhang, M. , Mujumdar, A. S. , & Zhu, Y. (2018). Emerging food drying technologies with energy‐saving characteristics: A review. Drying Technology, 37, 1465–1480.

[fsn33847-bib-0035] Hossain, M. A. , Mitra, S. , Belal, M. , & Zzaman, W. (2021). Effect of foaming agent concentration and drying temperature on biochemical properties of foam mat dried tomato powder. Food Research, 5, 291–297.

[fsn33847-bib-0036] Incoronato, A. L. , Cedola, A. , Conte, A. , & Del Nobile, M. A. (2021). Juice and by‐products from pomegranate to enrich pancake: Characterisation and shelf‐life evaluation. International Journal of Food Science & Technology, 56, 2886–2894.

[fsn33847-bib-0037] Inthuja, J. , Mahendran, T. , & Jemziya, M. B. F. (2020). Quality characteristics and sensory evaluation of cabbage (*Brassica oleracea* L. var. *capitata*) and lime (*Citrus aurantiifolia*) ready to serve ts beverage. Bangladesh Journal of Agricultural Research, 45, 157–164.

[fsn33847-bib-0038] Jeevarathinam, G. , Pandiselvam, R. , Pandiarajan, T. , Preetha, P. , Balakrishnan, M. , Thirupathi, V. , & Kothakota, A. (2021). Infrared assisted hot air dryer for turmeric slices: Effect on drying rate and quality parameters. LWT ‐ Food Science and Technology, 144, 111258.

[fsn33847-bib-0086] Joudi‐Sarighayeh, F., Abbaspour‐Gilandeh, Y., Kaveh, M., & Hernández‐Hernández, J. L. (2022). The Optimization of the Physical–Thermal and Bioactive Properties of Pumpkin Slices Dried in a Hybrid Microwave–Convective Dryer Using the Response Surface Method. Agronomy, 12(10), 2291. https://doi.org/10.3390/agronomy12102291

[fsn33847-bib-0039] Kanha, N. , Regenstein, J. M. , & Laokuldilok, T. (2022). Optimization of process parameters for foam mat drying of black rice bran anthocyanin and comparison with spray‐ and freeze‐dried powders. Drying Technology, 40, 581–594.

[fsn33847-bib-0040] Kelebek, H. , & Selli, S. (2011). Characterization of phenolic compounds in strawberry fruits by RP‐HPLC‐DAD and investigation of their antioxidant capacity. Journal of Liquid Chromatography and Related Technologies, 34, 2495–2504.

[fsn33847-bib-0041] Kılıç, E. E. , & Çınar, İ. (2019). Convective hot airdrying characteristics of selected vegetables. International Advanced Researches and Engineering Journal, 3(1), 7–13.

[fsn33847-bib-0042] Kumar, G. , Kumar, N. , Prabhakar, P. K. , & Kishore, A. (2022). Foam mat drying: Recent advances on foam dynamics, mechanistic modeling and hybrid drying approach. Critical Reviews in Food Science and Nutrition, 63, 8275–8291. 35380483 10.1080/10408398.2022.2053061

[fsn33847-bib-0043] LaPanse, A. J. , Krishnan, A. , & Posewitz, M. C. (2021). Adaptive laboratory evolution for algal strain improvement: Methodologies and applications. Algal Research, 53, 102122.

[fsn33847-bib-0044] Leahu, A. , Ghinea, C. , Oroian, M.‐A. , & Damian, C. (2018). Determination of essential and toxic elements, ascorbic acid content and color of different leaves in two cabbage varieties. Ovidius University Annals of Chemistry, 29, 110–116.

[fsn33847-bib-0045] Leão, D. P. , Franca, A. S. , Oliveira, L. S. , Bastos, R. , & Coimbra, M. A. (2017). Physicochemical characterization, antioxidant capacity, total phenolic and proanthocyanidin content of flours prepared from pequi (*Caryocar brasilense* Camb.) fruit by‐products. Food Chemistry, 225, 146–153. 28193408 10.1016/j.foodchem.2017.01.027

[fsn33847-bib-0046] Lobo, F. A. , Nascimento, M. A. , Domingues, J. R. , Falcão, D. Q. , Hernanz, D. , Heredia, F. J. , & de Lima Araujo, K. G. (2017). Foam mat drying of Tommy Atkins mango: Effects of air temperature and concentrations of soy lecithin and carboxymethylcellulose on phenolic composition, mangiferin, and antioxidant capacity. Food Chemistry, 221, 258–266. 27979201 10.1016/j.foodchem.2016.10.080

[fsn33847-bib-0047] Maciel, K. S. , Teixeira, L. J. Q. , Lucia, S. M. D. , & Saraiva, S. H. (2022). Optimization of foam mat drying for instant coffee processing and its effect on drying kinetics and quality characteristics. Drying Technology, 40, 1866–1880.

[fsn33847-bib-0048] Martín‐García, B. , Pimentel‐Moral, S. , Gómez‐Caravaca, A. M. , Arráez‐Román, D. , & Segura‐Carretero, A. (2020). Box‐Behnken experimental design for a green extraction method of phenolic compounds from olive leaves. Industrial Crops and Products, 154, 112741.

[fsn33847-bib-0049] Marzuki, S. U. , Pranoto, Y. , Khumsap, T. , & Nguyen, L. T. (2021). Effect of blanching pretreatment and microwave‐vacuum drying on drying kinetics and physicochemical properties of purple‐fleshed sweet potato. Journal of Food Science and Technology, 58, 2884–2895. 34294950 10.1007/s13197-020-04789-5 PMC8249554

[fsn33847-bib-0050] Mashau, M. E. , Mabodze, T. , Tshiakhatho, O. J. , Silungwe, H. , & Ramashia, S. E. (2020). Evaluation of the content of polyphenols, antioxidant activity and physicochemical properties of tortillas added with Bambara groundnut flour. Molecules, 25, 3035. 32635138 10.3390/molecules25133035 PMC7411947

[fsn33847-bib-0051] Mezzetti, B. , Biondi, F. , Balducci, F. , Capocasa, F. , Mei, E. , Vagnoni, M. , Visciglio, M. , & Mazzoni, L. (2022). Variation of nutritional quality depending on harvested plant portion of broccoli and black cabbage. Applied Sciences, 12, 6668.

[fsn33847-bib-0085] Motevali, A., Minaei, S., Khoshtaghaza, M. H., & Amirnejat, H. (2011). Comparison of energy consumption and specific energy requirements of different methods for drying mushroom slices. Energy, 36(11), 6433–6441. https://doi.org/10.1016/j.energy.2011.09.024

[fsn33847-bib-0052] Nisoa, M. , Wattanasit, K. , Tamman, A. , Sirisathitkul, Y. , & Sirisathitkul, C. (2021). Microwave drying for production of rehydrated foods: A case study of stink bean (*Parkia speciosa*) seed. Applied Sciences, 11, 2918.

[fsn33847-bib-0053] Nunes, M. C. N. , & Delgado, A. (2014). Quality of organic compared to conventionally grown strawberries at the retail level. Acta Horticulturae, 1049, 723–730.

[fsn33847-bib-0054] Ornelas‐Paz, J. D. J. , Yahia, E. M. , Ramírez‐Bustamante, N. , Pérez‐Martínez, J. D. , Escalante‐Minakata, M. D. P. , Ibarra‐Junquera, V. , Acosta‐Muñiz, C. , Guerrero‐Prieto, V. , & Ochoa‐Reyes, E. (2013). Physical attributes and chemical composition of organic strawberry fruit (*Fragaria* x *ananassa* Duch, Cv. *Albion*) at six stages of ripening. Food Chemistry, 138, 372–381. 23265501 10.1016/j.foodchem.2012.11.006

[fsn33847-bib-0055] Özbakir Özer, M. , Kar, H. , Murat Dogru, S. , & Kobal Bekar, N. (2021). Morphological characterization of some hybrid red head cabbage (*Brassica oleracea* L. var. *capitata* subvar. *rubra*) varieties. Journal of Tekirdag Agriculture Faculty, 18, 428–435.

[fsn33847-bib-0056] Ozcan‐Sinir, G. , Ozkan‐Karabacak, A. , Tamer, C. E. , & Copur, O. U. (2019). The effect of hot air, vacuum and microwave drying on drying characteristics, rehydration capacity, color, total phenolic content and antioxidant capacity of kumquat (*Citrus japonica*). Food Science and Technology, 39, 475–484.

[fsn33847-bib-0057] Ozcelik, M. , Ambros, S. , Morais, S. I. F. , & Kulozik, U. (2020). Storage stability of dried raspberry foam as a snack product: Effect of foam structure and microwave‐assisted freeze drying on the stability of plant bioactives and ascorbic acid. Journal of Food Engineering, 270, 109779.

[fsn33847-bib-0058] Ozcelik, M. , Heigl, A. , Kulozik, U. , & Ambros, S. (2019). Effect of hydrocolloid addition and microwave‐assisted freeze drying on the characteristics of foamed raspberry puree. Innovative Food Science & Emerging Technologies, 56, 102183.

[fsn33847-bib-0059] Ozcelik, M. M. , Ozkan, G. , & Karacabey, E. (2022). Evaluation of carbonic maceration effect as a pre‐treatment on the drying process of strawberry. Agriculture, 12(12), 2113.

[fsn33847-bib-0084] Özkan Karabacak, A. (2023). Assessment of Total Phenolic Compounds, Antioxidant Capacity, β‐Carotene Bioaccessibility, HMF Formation, and Color Degradation Kinetics in Pumpkin Pestils. *Journal of the Turkish Chemical Society Section A: Chemistry*, 10(3), 729–744. https://doi.org/10.18596/jotcsa.1302567

[fsn33847-bib-0060] Podsedek, A. , Majewska, I. , & Kucharska, A. Z. (2017). Inhibitory potential of red cabbage against digestive enzymes linked to obesity and type 2 diabetes. Journal of Agricultural and Food Chemistry, 65, 7192–7199. 28753316 10.1021/acs.jafc.7b02499

[fsn33847-bib-0061] Prabhanjan, D. G. , Ramaswamy, H. S. , & Raghavan, G. S. V. (1995). Microwave‐assisted convective air drying of thin layer carrots. Journal of Food Engineering, 25, 283–293.

[fsn33847-bib-0062] Putra, R. N. , & Ajiwiguna, T. A. (2017). Influence of air temperature and velocity for drying process. Procedia Engineering, 170, 516–519.

[fsn33847-bib-0063] Qadri, O. S. (2022). Microwave drying of foamed tomato pulp: Optimization and mass transfer modelling. Journal of Food Processing and Preservation, 46, e15954.

[fsn33847-bib-0064] Qadri, O. S. , Osama, K. , & Srivastava, A. K. (2020). Foam mat drying of papaya using microwaves: Machine learning modeling. Journal of Food Process Engineering, 43, e13394.

[fsn33847-bib-0065] Qadri, O. S. , & Srivastava, A. K. (2017). Microwave‐assisted foam mat drying of guava pulp: Drying kinetics and effect on quality attributes. Journal of Food Process Engineering, 40, e12295.

[fsn33847-bib-0066] Qadri, O. S. , & Srivastava, A. K. (2021). Prototype continuous microwave foam‐mat dryer: Design and fabrication. Journal of Food Science and Technology, 58, 3357–3367. 34366453 10.1007/s13197-020-04907-3 PMC8292499

[fsn33847-bib-0067] Rajkumar, P. , Kailappan, R. , Viswanathan, R. , & Raghavan, G. S. V. (2007). Drying characteristics of foamed alphonso mango pulp in a continuous type foam mat dryer. Journal of Food Engineering, 79, 1452–1459.

[fsn33847-bib-0068] Reis, F. R. , de Moraes, A. C. S. , & Masson, M. L. (2021). Impact of foam‐mat drying on plant‐based foods bioactive compounds: A review. Plant Foods for Human Nutrition, 76, 153–160. 34052949 10.1007/s11130-021-00899-3

[fsn33847-bib-0069] Roknul Azam, S. M. , Zhang, M. , Law, C. L. , & Mujumdar, A. S. (2019). Effects of drying methods on quality attributes of peach (*Prunus persica*) leather. Drying Technology, 37, 341–351.

[fsn33847-bib-0070] Rzepecka, M. A. , Brygidyr, A. M. , & McConnell, M. B. (1976). Foam‐mat dehydration of tomato paste using microwave energy. Canadian Agricultural Engineering, 18, 36–40.

[fsn33847-bib-0071] Sarkar, A. , Rahman, S. , Roy, M. , Alam, M. , Hossain, M. A. , & Ahmed, T. (2021). Impact of blanching pretreatment on physicochemical properties, and drying characteristics of cabbage (*Brassica oleracea*). Food Research, 5, 393–400.

[fsn33847-bib-0072] Shaari, N. A. , Sulaiman, R. , Rahman, R. A. , & Bakar, J. (2018). Production of pineapple fruit (*Ananas comosus*) powder using foam mat drying: Effect of whipping time and egg albumen concentration. Journal of Food Processing and Preservation, 42, e13467.

[fsn33847-bib-0073] Shankar, S. , Segaran, G. , Sundar, R. D. V. , Settu, S. , & Sathiavelu, M. (2019). Brassicaceae‐A classical review on its pharmacological activities. International Journal of Pharmaceutical Sciences Review and Research, 55, 107–113.

[fsn33847-bib-0074] Sramek, M. , Schweiggert, R. M. , Van Kampen, A. , Carle, R. , & Kohlus, R. (2015). Preparation of high‐grade powders from tomato paste using a vacuum foam drying method. Journal of Food Science, 80, E1755–E1762. 26189747 10.1111/1750-3841.12965

[fsn33847-bib-0075] Sun, Y. , Zhang, Y. , Xu, W. , & Zheng, X. (2020). Analysis of the anthocyanin degradation in blue honeysuckle berry under microwave assisted foam‐mat drying. Food, 9, 397. 10.3390/foods9040397 PMC7231185 32244338

[fsn33847-bib-0076] Szulc, K. , & Lenart, A. (2016). Effect of composition on physical properties of food powders. International Agrophysics, 30(2), 237–243.

[fsn33847-bib-0077] Tatar, F. , & Kahyaoglu, T. (2015). Microencapsulation of anchovy (*Engraulis encrasicolus* L.) oil: Emulsion characterization and optimization by response surface methodology. Journal of Food Processing and Preservation, 39(6), 624–633.

[fsn33847-bib-0078] Varhan, E. , Elmas, F. , & Koç, M. (2019). Foam mat drying of fig fruit: Optimization of foam composition and physicochemical properties of fig powder. Journal of Food Process Engineering, 42, e13022.

[fsn33847-bib-0079] Wojdyło, A. , Figiel, A. , & Oszmiański, J. (2009). Effect of drying methods with the application of vacuum microwaves on the bioactive compounds, color, and antioxidant activity of strawberry fruits. Journal of Agricultural and Food Chemistry, 57, 1337–1343. 19170638 10.1021/jf802507j

[fsn33847-bib-0080] Yüksel, A. N. (2021). Development of yoghurt powder using microwave‐assisted foam‐mat drying. Journal of Food Science and Technology, 58, 2834–2841. 34194117 10.1007/s13197-021-05035-2 PMC8196149

[fsn33847-bib-0081] Zayed, A. , Sheashea, M. , Kassem, I. A. A. , & Farag, M. A. (2022). Red and white cabbages: An updated comparative review of bioactives, extraction methods, processing practices, and health benefits. Critical Reviews in Food Science and Nutrition, 63, 7025–7042. 35174750 10.1080/10408398.2022.2040416

[fsn33847-bib-0082] Zielinska, M. , Ropelewska, E. , Xiao, H.‐W. , Mujumdar, A. S. , & Law, C. L. (2020). Review of recent applications and research progress in hybrid and combined microwave‐assisted drying of food products: Quality properties. Critical Reviews in Food Science and Nutrition, 60, 2212–2264. 31257908 10.1080/10408398.2019.1632788

