# Supplementary material for: Preserving nutrient content in red cabbage juice powder via foam‐mat hybrid microwave drying: Application in fortified functional pancakes
Source: Food Sci Nutr. 2023 Nov 20;12(2):1340–55. doi: 10.1002/fsn3.3847 (PMC10867499; doi:10.1002/fsn3.3847)
Supplement: Supplementary file 1 — Table S1 [file FSN3-12-1340-s001.docx]

**Suplementary Table 1.**

| OTP RCJP |
| --- |
| 0.80±0.17 |
| 190.38± 0.10 |
| 104.79±0.34 |
| 0.76±0.00 |
| 3.50±0.02 |
| 0.3±0.00 |
| 5.07±0.02 |
| 42.12±0.01 |
| 10.95±0.43 |
| 0.39±0.01 |
| 6.75±0.05 |
| 20±0.50 |
| 3.42±0.05 |
| 165.20±1.16 |
| 85.74±1.32 |
| 97.60±0.42 |
| 137.38±0.20 |

* FT: foam thickness in tray (mm), FC: foam capacity (%), FS: foam stability (%), BD: bulk density (%), WAI: water absorption index (%), WSI: water solubility index (%), SP: swelling power (%), SSC: total soluble solid content, DT: drying time (min), BI: browning index, TAC: total anthocyanin content (mg cyanidin3g/100g dm), AAC: ascorbic acid content (mg/100g dm), TEAC: trolox equivalent antioxidant capacity (mg/100g dm), TPC: total phenolic content (mg GAE/100g dm), MC: moisture content (%), WA: water activity (a_w_), OTP: outside the trial plan (EWP 12g/100g, CMC 0.2g/100g, 360W-100°C)
